# Neuronal processes contain the essential components for the late steps of ribosome biogenesis

**DOI:** 10.1073/pnas.2502424122

**Published:** 2025-07-31

**Authors:** Claudia M. Fusco, Anja Staab, Ashley M. Bourke, Georgi Tushev, Kristina Desch, Erico Moreto Lins, Elena Ciirdaeva, Susanne tom Dieck, Nina Kaltenschnee, Alexander Heckel, Julian D. Langer, Erin M. Schuman

**Affiliations:** ^a^Department of Synaptic Plasticity, Max Planck Institute for Brain Research, Frankfurt 60438, Germany; ^b^Institute for Organic Chemistry and Chemical Biology, Goethe-University Frankfurt, Frankfurt 60438, Germany

**Keywords:** ribosome, local translation, synapses, protein synthesis

## Abstract

Neurons rely on spatial and temporal control of protein synthesis to respond rapidly and locally to external stimuli, a process facilitated by the dynamic localization and modification of ribosomes. While previous research has shown that neuronal activity can regulate ribosome localization and modify translation rates, little is known about ribosomal assembly within neuronal processes. Here, we investigated the potential for local ribosome maturation in rat neurons using proteomics, RNA sequencing, and imaging methods. We detected an abundance of ribosome biogenesis factors in distal neuronal compartments, particularly those associated with the late stages of ribosome assembly. Moreover, we detected cytosolic pre-ribosomal RNA species in dendrites, as well as the enzymes necessary for their processing, suggesting that local ribosome maturation can occur far from the nucleus. These findings challenge conventional models that confine ribosome biogenesis to nuclear and perinuclear regions and suggest that neurons may fine-tune local protein synthesis by regulating ribosome assembly near synaptic sites. This mechanism may enable rapid modulation of the translational capacity in response to physiological changes, regulating synaptic plasticity and local protein synthesis in neurons.

## Significance

Neurons require precise spatial and temporal regulation of protein synthesis to adapt rapidly to external stimuli, particularly at synapses. Our study challenges the view that new ribosomes are made exclusively near the nucleus and reveals that ribosome biogenesis factors and pre-ribosomal RNAs are present in distal neuronal compartments. These findings suggest that ribosome maturation can occur locally in dendrites, enabling rapid, spatially targeted modulation of translational capacity. This mechanism provides a different framework for understanding how neurons regulate synaptic plasticity and adapt to physiological changes. By demonstrating that ribosome assembly may extend beyond the nucleus, this work highlights a additional layer of neuronal protein synthesis control, potentially transforming our understanding of how neurons orchestrate local responses to environmental cues.

All cells adapt to their environment through dynamic adjustments to their proteome. Neurons, with their extensive spatial compartmentalization and long lifespan, use a variety of mechanisms to regulate the spatial and temporal availability of proteins. A critical aspect of this regulation is the distribution of the translational machinery (ribosomes) within neuronal processes (1–5), linking protein synthesis directly to neuronal activity and ensuring rapid, localized responses to external stimuli (6, 7).

Neurons also fine-tune local protein synthesis by regulating the local messenger RNA (mRNA) pool and modifying the activity of translation factors (8–11). Importantly, the ribosome itself can also be a target of regulation (12). For example, neuronal activity leads to the phosphorylation of Ribosomal Protein S6, affecting the translation of specific mRNAs (13, 14). Additionally, increased production of ribosomal proteins (RPs) and the resulting altered ribosome composition leads to the impaired translational homeostasis observed in neurodevelopmental disorders, like the Fragile X syndrome (15). Furthermore, neurons can enhance their local translation capacity in response to activity, by regulating ribosome trafficking (16) and changing their localization—for example—from dendritic shafts to spines or from granular to free states (2, 17). Relatively little is known, however, about whether neuronal processes possess the capacity for core compositional rearrangements of local ribosomes.

The mRNAs encoding RPs have also been observed in neuronal processes (18–24). We and others have shown that RPs synthesized within neurites (dendrites and axons) can associate with local, preexisting ribosomes (25, 26). This dynamic RP incorporation could be used for ribosome repair and/or specialization. In fact, the RP incorporation probability depends on the subcellular location and physiological state. For example, the incorporation of a number of nascent RPs was increased after oxidative stress (25) or after stimulation with a growth factor (26). Similar findings have recently been observed in yeast, where one of the exchanging RPs (RPS26) gets damaged following oxidative stress, and replaced by a new functional copy (27, 28). During this process, ribosomes that transiently lack RPS26 acquire a new translational preference for genes related to the stress-response (29). Neurons may use similar mechanisms to dynamically modify the translational machinery near synapses. Additionally, there is the possibility that distally synthesized RPs can be incorporated into premature ribosomes, facilitating their maturation and thereby expanding the local pool of functional ribosomes. This idea of local ribosome maturation, however, challenges the prevailing view that immature ribosomal particles are only found in nuclear and perinuclear regions.

Ribosome biogenesis begins with ribosomal RNA (rRNA) transcription in the nucleolus and involves the coordinated association of several ribosome biogenesis factors (RBFs), RPs, and small nucleolar RNAs (snoRNAs). Such interactions allow pre-rRNA processing (cleavage), modification (pseudouridylation and methylation), and folding into preribosomal subunits (30). This process continues in the nucleoplasm and ends in the cytoplasm, where the small (40S) and large (60S) ribosomal subunits become competent for mRNA translation (31). Although the overall process is highly conserved, the discovery of unique regulatory features in mammalian cells has highlighted a more complex assembly pathway in higher eukaryotes (32). Most RBFs transiently or stably associate with nascent preribosomal particles and trigger conformational rearrangements at specific steps either in the nucleolus, nucleoplasm, or cytoplasm. Maturation is then completed when all RBFs are removed and all RPs are incorporated (32). Throughout the process, several quality control mechanisms ensure the proper folding and functional competence of mature ribosomes (33). However, whether ribosome biogenesis, particularly its later stages, is modulated by physiological conditions, remains poorly understood (34). Instead, most research has focused on the regulation of the early steps, such as rRNA transcription and RP translation (15), often in relation to cellular proliferation and cancer through pathways like mTOR signaling (35, 36).

Here, we used imaging, mass spectrometry (MS), and RNA sequencing to characterize markers of ribosome biogenesis in different neuronal subcellular compartments. We found that particles from the late (but not early) stages of ribosome biogenesis are localized in distal neuronal processes. Our findings suggest a mechanism by which neurons may modify their translation capacity through the local maturation of ribosomes.

## Results

### The Subcellular Distribution of Translation-Related Proteins and RBF.

**Fig. 1.**
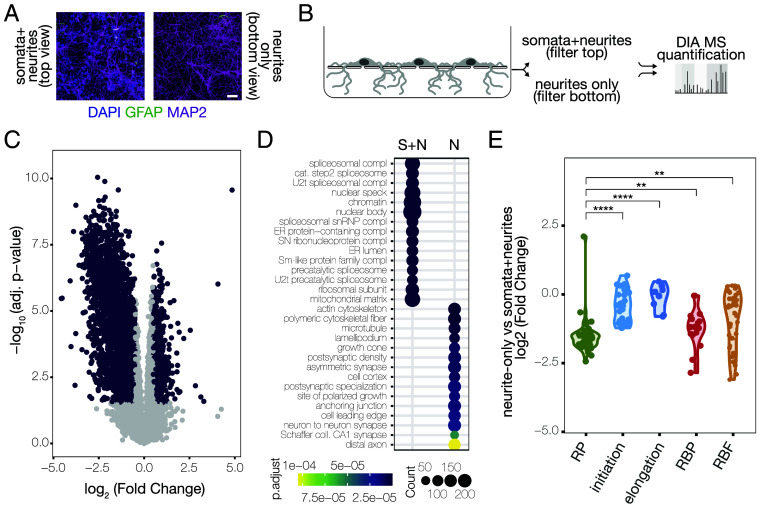
Local proteome profiling reveals the subcellular distribution of translation-related proteins. (*A*) Representative images indicating the presence of dendrites (anti-MAP2; *Top* and *Bottom*), nuclei (DAPI; *Top* only), and glia (anti-GFAP; *Top* only) in the top or bottom sections of a compartmentalized chamber, as indicated (Scale bar, 50 μm). (*B*) Schematic of the experimental design for measuring protein abundance between subcellular compartments. The top and bottom of each filter were separately harvested for downstream DIA MS. (*C*) Volcano plot of significantly regulated proteins (purple, FDR < 0.05 and >|0.5|log-scaled fold change) between total lysates from the somata + neurite and neurites-only fractions. Unpaired, two-sided *t* test with Welch correction on rows. Benjamini–Hochberg correction for multiple testing. 2,594 proteins were significantly enriched in the somata + neurites fraction, 944 in neurites only. (*D*) GO analysis of Cellular Components terms overrepresented (FDR < 0.01) among the differentially regulated proteins between total lysates from soma+neurite (S + N) and neurites-only (N). Abbreviations: compl (complex), cat. (catalytic), ER (endoplasmic reticulum), SN (small nuclear), U2t (U2-type), coll. (collateral). (*E*) Violin plot of the log2 fold change of the protein abundance between neurite-only and soma+neurite fractions, grouped by gene category. The horizontal line represents log2 fold change = 0. Above the line, proteins were enriched in the neurite-only fraction. Below the line, proteins were enriched in soma+neurite fraction. RP (n = 47), initiation (n = 29), elongation (n = 8), RBP (n = 16), RBF (n = 36). Each dot represents a gene of interest. Kruskal−Wallis, *P* < 2.2e−16; *t* test between RP and initiation, *P* = 1.8e−14; *t* test between RP and elongation, *P* = 2.2e−07; *t* test between RP and RBP (RNA Binding Proteins), *P* = 0.004; *t* test between RP and RBF (Ribosome Biogenesis Factors), *P* = 0.0017.

To characterize, in an unbiased manner, the distribution of translation-related proteins between subcellular compartments, we cultured rat cortical neurons in compartmentalized chambers that allow for the enrichment of a neurite (axon + dendrite) fraction for comparison with a mixed population of somata and neurites (Fig. 1*A*). We then separately purified proteins from either compartment and used Data Independent Acquisition (DIA) Mass Spectrometry (MS) to characterize the subcellular proteomes (Fig. 1*B*). We reliably identified ~9,000 and ~8,000 protein groups in the somata+neurite and neurite-only samples, respectively (*SI Appendix*, Fig. S1). We compared the proteome from these two compartments and found 2,594 proteins enriched in the somata + neurite fraction and 944 proteins enriched in the neurite-only fraction (Fig. 1*C*). Gene ontology (GO) analysis of the differentially regulated proteins confirmed the specificity of our preparation: Terms associated with the nucleus or the perinuclear region [e.g., the spliceosome and Endoplasmic Reticulum (ER), respectively] were enriched in the somata + neurite fraction, while synaptic terms were enriched in the neurite-only fraction (Fig. 1*D*). To examine how neurons distribute their translational machinery between subcellular compartments, we curated a list of gene categories related to protein synthesis including RPs, initiation factors, elongation factors, RNA binding proteins, and RBFs. We determined the degree to which a given category was enriched in either compartment, relative to the other. When analyzed in this way, RPs showed a strong de-enrichment in neurites and a strong enrichment in somata + neurites (Fig. 1 *E* and *D*). Interestingly, initiation and elongation factors were, in contrast, equally distributed between compartments (Fig. 1*E*). These two observations are consistent with the recent observation that protein synthesis in the cell body is mostly undertaken by polysomes (several ribosomes translating the same mRNA), while neuronal processes tend to prefer protein synthesis by monosomes (one single ribosome per mRNA) (19). The higher ratio of translation factors per ribosome in the neurite compartment fits with the slower initiation and elongation rates exhibited by monosome-preferring transcripts, as they require more time to process a higher number of secondary structures in the mRNA (19). This is paralleled by the apparent higher proportion of RNA binding proteins per ribosome in the neurite compartment (Fig. 1*E*). Altogether, our data suggest that neuronal processes have a high capacity to regulate RNA localization and translation by differentially distributing key regulators of protein synthesis.

**Fig. 2.**
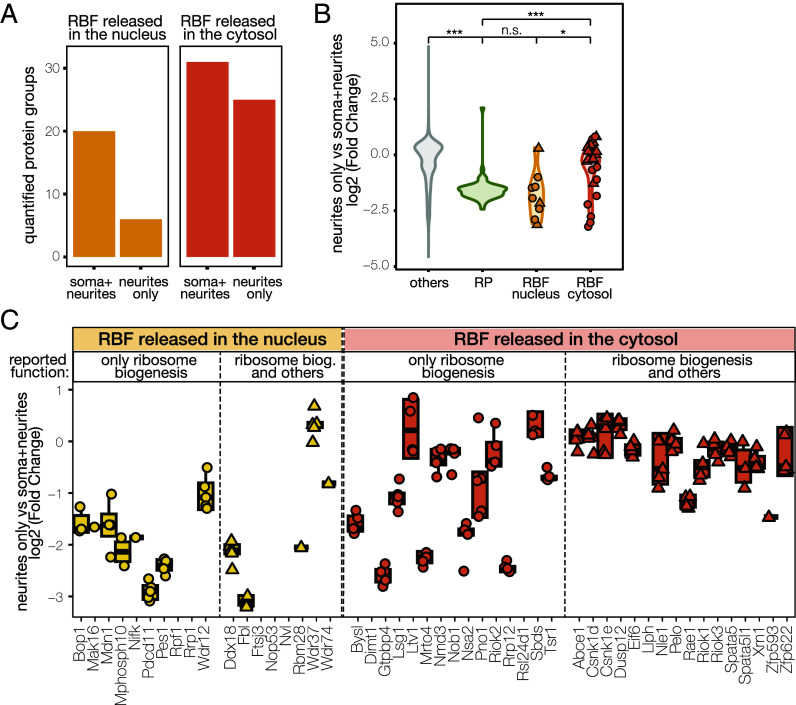
Cytosolic (but not nuclear) RBFs localize to neuronal processes. (*A*) Bar plot of the number of identified RBFs in all biological replicates (5/5) of each subcellular compartment. The bars related to RBFs that are released in the nucleus are colored light orange, and those for RBFs that are released in the cytoplasm in dark orange. (*B*) Violin plot of the log2 fold change of the protein abundance between neurite-only and somata + neurite fractions, as in (*A*). Each dot represents an RBF. The shape of the dot indicates whether the corresponding protein is known to have other (e.g., moonlighting) functions. RBFs that dissociate from the preribosome subunits in the cytosol (in orange, n = 27), but not those that are released in the nucleus (in yellow, n = 9), showed a significantly higher proportion in the neurite-only fraction compared to RPs (in green, n = 47). Kruskal−Wallis, *P* < 2.2e−16; *t* test between RP and others, *P* < 2.22e−16; *t* test between RP and RBF nucleus, *P* = 0.32; *t* test between RP and RBF cytosol, *P* = 2.6e−06; *t* test between RBF nucleus and RBF cytosol, *P* = 0.0098. (*C*) Boxplot showing the log2 intensity of quantified RBFs in neurites only, compared to the soma+neurite compartment. Each point represents a biological replicate. Proteins quantified in one compartment only are shown as missing values. RBFs that are released in the nucleus are yellow, and those for RBFs that are released in the cytoplasm in orange. The shape of the dot indicates if the corresponding protein is known to have extraribosomal (e.g., moonlighting) functions. Abbreviations: Ribosomal Proteins (RP), RNA Binding Protein (RBP), Ribosome Biogenesis Factors (RBF), repl. (biological replicate).

The above proteomic characterization also revealed a surprisingly high proportion of RBFs in the neurite compartment (Fig. 1*E*). To explore this further, we asked whether the detection and abundance of an RBF in the neurite compartment is influenced by the location (nuclear vs. cytoplasmic) in which it is known to interact with immature ribosomes. Indeed, most RBFs that dissociate from the preribosomal subunits in the nucleus were detected only in the somata + neurite compartment. In contrast, almost all the RBFs that dissociate from the presubunits in the cytoplasm were detected in both compartments (Fig. 2*A*). Importantly, when detected in the neurites, nuclear RBFs displayed lower levels than their cytoplasmic counterparts (Fig. 2 *B* and *C* and *SI Appendix*, Fig. S1 *D*–*F*).

**Fig. 3.**
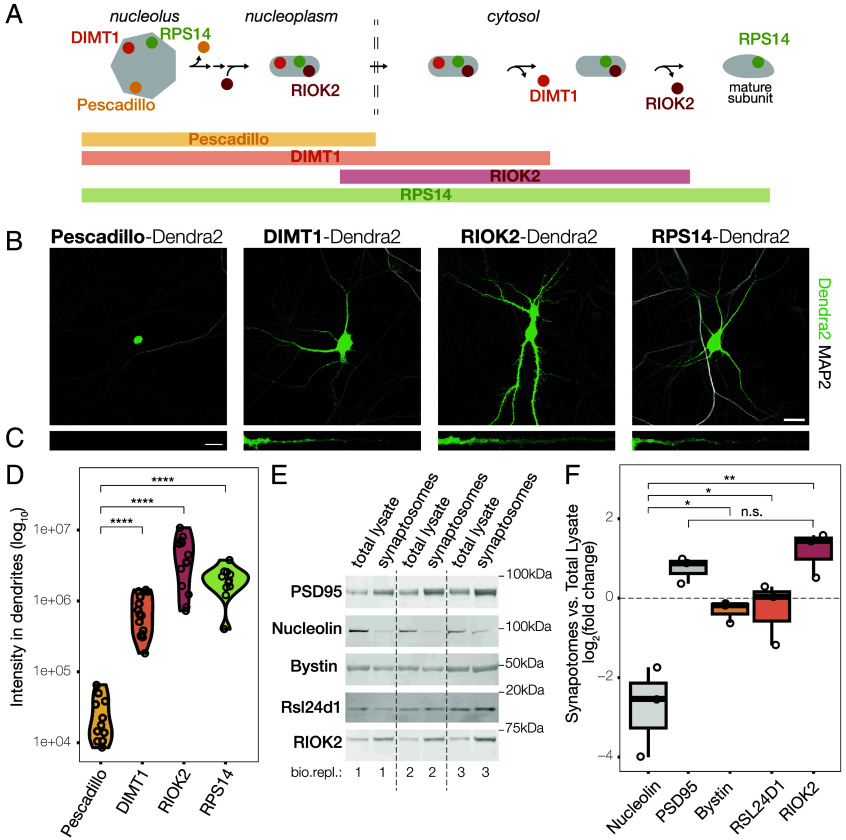
Cytosolic (but not nuclear) RBFs localize to neuronal processes and synapses. (*A*) Schematic representation of the incorporation and dissociation of different biogenesis factors (Pescadillo, DIMT1, RIOK2, respectively in yellow, orange, and red) and a Ribosomal Protein (RPS14, in green) in different cellular compartments (from left to right: nucleolus, nucleoplasm, and cytosol) during the maturation of the ribosomal subunits (in gray). (*B*) Representative images showing the localization (within individual neurons) of different biogenesis factors (Pescadillo, DIMT1, RIOK2) and a Ribosomal Protein (RPS14) fused with the common fluorescent reporter Dendra2 (Scale bar, 25 μm). The dendritic marker MAP2 is shown in gray, Dendra2 in green. (*C*) Straighten dendrites of neurons shown in Fig. 3*B* (Scale bar, 10 μm). All dendrites are shown with the same orientation (part most proximal to the soma is on the left, distal to the right). The Dendra2 channel is shown in green. (*D*) Violin plot depicting the Dendra2 intensity of the indicated proteins in the dendritic arbor of individual neurons. The number of imaged cells was between 11 and 14 (as indicated by the dot plots), over three independent experiments. Kruskal−Wallis, *P* = 4.3e−08; *t* test between Pescadillo and DIMT1, *P* = 4.5e−07; *t* test between Pescadillo and RIOK2, *P* = 8e−07; *t* test between Pescadillo and RPS14, *P* = 2.8e−06. (*E*) Detection of proteins-of-interest in total lysate and synaptosome fractions from the rat cortex by western blot (three biological replicates). (*F*) Quantification of western blot signals, showing the log_2_ fold change in normalized protein levels (relative to total protein staining) between synaptosome and total lysate fractions. The nuclear marker Nucleolin was strongly depleted in synaptosomes, while the synaptic marker PSD95 was enriched, confirming the purity of the preparation. All three tested RBFs—RSL24D1, Bystin, and RIOK2—were present in synaptosomes at levels exceeding those of Nucleolin (ANOVA, *P* = 0.00038; paired *t* test: ENP1, *P* = 0.043; RSL24D1, *P* = 0.019; RIOK2, *P* = 0.0077). The enrichment of RIOK2 in synaptosomes was not significantly different from that of PSD95 (paired *t* test, *P* = 0.43). Abbreviation: Pes (Pescadillo).

To validate the unexpected detection of RBFs in neuronal processes, we created fluorescent protein fusion constructs and assessed their subcellular localization following expression in neurons. We selected three RBFs and one RP that participate in the maturation of ribosomal subunits at well-characterized checkpoints and for which no other function besides ribosome biogenesis has been reported (Fig. 3*A*). As a “nuclear only” control, we chose the protein Pescadillo which is known to incorporate into pre-60S particles in the nucleolus and dissociate once in the nucleoplasm (37). As cytoplasmic RBFs, we chose DIMT1 and RIOK2. DIMT1 associates with the small subunit processome in the nucleolus and is released in the cytoplasm (38, 39). RIOK2 is incorporated into the pre–small subunit just prior to nuclear export and is one of the last factors to be released in the cytoplasm as biogenesis is completed (32). Finally, as a constitutive element of ribosomes, we chose Ribosomal Protein RPS14, which stably associates with the ribosome early (in the nucleolus) and remains a core element of the small ribosomal subunit for its lifespan (40). To avoid difficulties that might arise from differences in antibody–epitope binding, we fused each protein of interest to the fluorescent reporter Dendra2 and transfected cultured neurons. One day after transfection, we imaged individual neurons and quantified the distribution of each protein-of-interest in the nucleus, and in the somatic and dendritic cytoplasm. As expected, Pescadillo was detected only in the nucleus (both nucleolus and nucleoplasm), while DIMT1, RIOK2, and RPS14 were also detected in the cytoplasm (Fig. 3*B*). Importantly, the cytoplasmic signal of both DIMT1 and RIOK2 was not confined to the soma but distributed throughout the dendritic arbor (Fig. 3 *C* and *D*). The signal for RIOK2 was especially prominent at putative synaptic sites.

To further assess the presence of RBFs at synapses, we analyzed synaptosome preparations from the rat cortex. Western blot analysis confirmed the enrichment and depletion of synaptic and nuclear material, as validated by the presence of the synaptic marker PSD95 and the absence of the nuclear marker Nucleolin, respectively. We then quantified three RBFs—RSL24D1, Bystin, and RIOK2—that associate sequentially with preribosomal particles in the nucleus and are later released into the cytosol (32). Importantly, none of these proteins have reported functions outside of ribosome biogenesis. All three RBFs were detected in the synaptosome fractions at levels exceeding those of nuclear markers, with RIOK2 reaching enrichment levels comparable to PSD95 (Fig. 3 *E* and *F*).

Altogether, our imaging and biochemical data corroborate the observation that cytosolic and late stage RBFs populate neuronal processes and synapses.

### RBFs from Neurites Are Assembled in Particles.

**Fig. 4.**
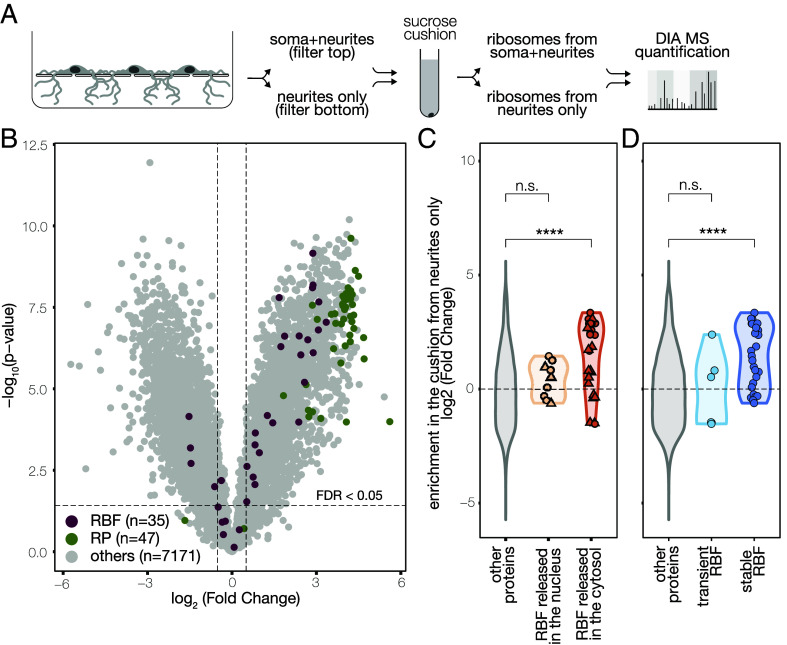
RBFs from neurites are assembled in particles. (*A*) Schematic of the experimental design. Immature and mature ribosomes were purified from either subcellular compartment (somata + neurites vs. neurites only) via sucrose cushioning and measured by MS. (*B*) Volcano plot of differentially abundant proteins between total lysates and cushion from neurites. Unpaired, two-sided *t* test with Welch correction on rows. Benjamini–Hochberg correction for multiple testing. (*C*) Violin plot of the log2 fold change of the protein abundance in the cushion vs. the total lysate, grouped by gene category. Each dot represents a protein-of- interest. RBFs that dissociate from the presubunits in the cytosol (in orange, n = 26), but not those that are released in the nucleus (in yellow, n = 9), were significantly enriched in the cushion from neurites. Kruskal−Wallis, *P* = 0.00014; *t* test between others and RBF nucleus, *P* = 0.29; *t* test between others and RBF cytosol, *P* = 4.6e−05. The shape of the dot indicates whether the corresponding protein is known to have (triangles) or not have (circles) other (e.g., moonlighting) functions. (*D*) Violin plot of the log2 fold change of the protein abundance in the cushion vs. the total lysate, grouped by gene category. Each dot represents a protein-of-interest. RBFs that stably associate with presubunits (in dark blue, n = 27), but not those that are just transient interactors (in light blue, n = 7) were significantly enriched in the cushion from neurites. Kruskal−Wallis, *P* = 7.5e−05; *t* test between others and RBF nucleus, *P* = 0.97; *t* test between others and RBF cytosol, *P* = 1.3e−05.

The RBFs we observed in dendrites have not been reported to have any other function outside ribosome biogenesis (Figs. 2 and 3 and *SI Appendix,* Fig. S1 *D*–*F*); as such, the dendritic localization of these proteins suggests the presence of premature ribosomes in distal processes. To examine this further, we asked whether RBFs in dendrites are associated with assembled ribosomal particles. To this end, we performed a sucrose cushion from either compartment (somata + neurites vs. neurite-only) and measured the proteins present by DIA MS (Fig. 4*A*). The sucrose cushion conditions were optimized to enrich not only actively translating ribosomes but also preribosomal particles (*SI Appendix*, Fig. S2; see *Materials and Methods*). In the neurite-only compartment, we reliably quantified 47 RPs which shared a homogenous and strong enrichment in the cushion, confirming the successful ribosomal purification. We also quantified 34 RBFs which showed a broader distribution (Fig. 4*B*). We asked whether the localization and strength of interaction between the RBF and the preribosomal particle could explain the breadth of distribution. Indeed, cytosolic, but not nuclear, RBFs and stable, but not transient ribosome interactors, were enriched in the cushion (Fig. 4 *C* and *D*). Altogether, our data show that RBFs in neuronal processes are stable components of assembled particles.

### Cytosolic (But Not Nuclear) Pre-rRNAs Localize to Neuronal Processes.

**Fig. 5.**
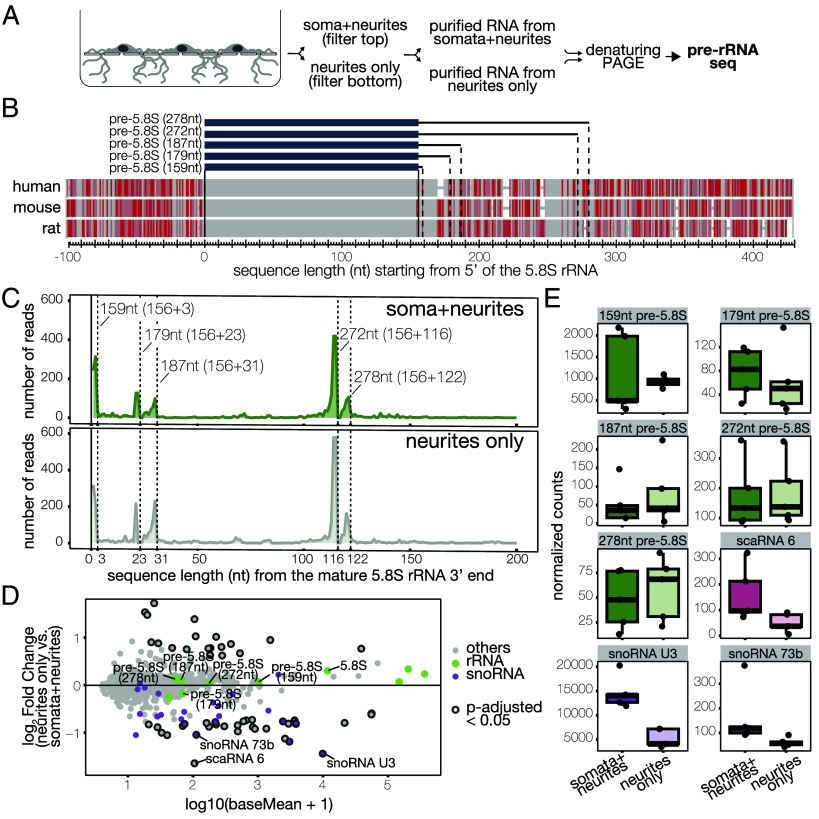
pre-rRNA-seq reveals cytosolic pre-5.8S rRNAs in rat neurons and neurites. (*A*) Multisequence alignment of the 100 nt upstream and 415 nt downstream of the first nucleotide of the mature 5.8S rRNA in the human, mouse, and rat. Identical nucleotides are shown in gray, mismatches in red according to the "Frequency-based differences" method of the NCBI Multiple Sequence Alignment Viewer, Version 1.25.1 (bases that occur more frequently are highlighted in lighter colors). Solid lines indicate the 5′ and 3′ ends of the mature 5.8S. Dashed lines indicate the predicted 3′ end of cytosolic pre-5.8S species identified. (*B*) Schematic of the experimental design. RNAs were purified from either subcellular compartment (somata + neurites vs. neurites only) and run on a denaturing TBE-Urea gel to separate RNAs of different sizes. RNA molecules were measured by RNAseq. (*C*) Coverage plot of the 3′ nucleotide of sequenced RNA molecules downstream of the mature 5.8S rRNA. The solid line indicates the 3′ end of the mature 5.8S. Dashed lines indicate the predicted 3′ end of the premature 5.8S molecules (estimate of the total length is indicated). On the top (dark green) reads in the somata + neurites sample. On the bottom (light green) reads in the neurite-only sample. Each coverage plot represents the sum of five biological replicates. (*D*) MA plot of the expression levels of RNAs identified in the somata+neurites and neurite-only compartments. SnoRNAs are shown in magenta, rRNAs in green. RNAs that were significantly enriched (adjusted *P*-value < 0.05) in either compartment are highlighted with a dark circle. RNAs of interest are indicated by name. (*E*) Box plot of count of reads mapped to the unique sequence of the cytosolic pre-5.8S molecules, small Cajal-body specific RNA (scaRNA 6), small nucleolar RNA (snoRNA) U3, and 73b, as indicated. Darker colors are used for the somata + neurites samples, lighter colors for the neurite-only samples. Each dot represents a biological replicate.

To examine whether these RBF-containing particles are indeed preribosomal subunits, we probed the localization of pre-rRNAs. The rDNA gene is initially transcribed as a long transcript (47S rRNA); the activity of exo- and endonucleases generates smaller intermediate molecules, until the 5.8S, 18S, and 28S rRNAs are fully matured and competent for translation in the cytosol. Studies from yeast and humans have revealed that major steps of ribosome biogenesis and rRNA processing are relatively conserved throughout evolution: The processing of the 28S rRNA occurs only in the nucleus, while the last steps of 18S and 5.8S rRNA maturation are completed in the cytosol. Despite the astonishingly high degree of conservation between mature rRNAs, the primary sequence around these regions is not conserved, even just within mammalian species (30) (Fig. 5*B*). We note that detecting these cytosolic precursors in neurons poses significant challenges, requiring methods with i) high sensitivity to detect signals in subcellular compartments with low RNA concentrations, ii) a high dynamic range to distinguish them from the highly concentrated signal in the nucleus, and iii) high resolution to differentiate mature and immature rRNA species which differ by only a few dozen nucleotides at their 3′ end. We tested several approaches, including fluorescence in situ hybridization, molecular beacons, and PCR without consistent success. As indicated below, highly sensitive Northern blotting and RNA sequencing proved successful for detecting cytosolic pre-5.8S rRNA, although detection of cytosolic pre-18S rRNA remained unsuccessful.

To identify the sequence of cytosolic pre-5.8S and predict their cleavage sites in rat tissue, we developed a highly sensitive pre-rRNA sequencing method (Fig. 5*A* and *SI Appendix,* Fig. S3*A*). Total RNA from rat neurons was analyzed using gel electrophoresis to separate RNA molecules according to their size. We then cut gel bands between ~160 nt and ~400 nt to enrich for cytosolic pre-5.8S species and de-enrich for mature 5.8S. Purified RNA was then processed to generate roughly 80 nt long reads covering the 3′ end of the sequence. As the difference between the mature 5.8S and its cytosolic preintermediates is at the 3′ end, the 3′ bias of our sequencing pipeline allowed for the prediction of the exact sequence length and potential cleavage sites of the pre-5.8S species. Additionally, the method was designed to overcome modifications and tertiary structures in the RNA, which are common for rRNA molecules but inhibit most conventional cDNA synthesis pipelines. Consistent with its abundance, the great majority of the reads mapped to the last 80 nt of the mature 5.8S rRNA sequence. However, we also quantified a significant number of reads that mapped to the 5′ end of the Internal Transcribed Spacer ITS2 and therefore were unique to pre-5.8S molecules (Fig. 5*C*). In total, we identified five pre-5.8S molecules (159, 179, 187, 272, and 278 nt long), three more than what has previously been identified in humans (180 nt and 325 nt long). Interestingly, when we compared the sequence of the cytosolic pre-5.8S between the human, mouse, and rat, we observed higher homology at the 5′ of the predicted cleavage sites in rat (Fig. 5*B*), suggesting that despite the overall low similarity in the primary sequence, the general processing and the responsible enzymes might be conserved across mammalian species. We then examined whether these pre-rRNA species can also be found specifically in neuronal processes. To do so, we applied our pre-rRNAseq method to the compartmentalized chambers described above, and identified the above pre-rRNA molecules in the neurite-only compartment, at levels similar to that detected in the somata + neurite compartment (Fig. 5 *D* and *E*). Importantly, nuclear RNAs like the small Cajal body-specific RNA 6 (scaRNA6) and the small nucleolar RNAs snoRNA U3 and snoRNA 73b, exhibited, instead, a specific de-enrichment in the neurite compartment, confirming the purity of our preparation (Fig. 5 *D* and *E*).

**Fig. 6.**
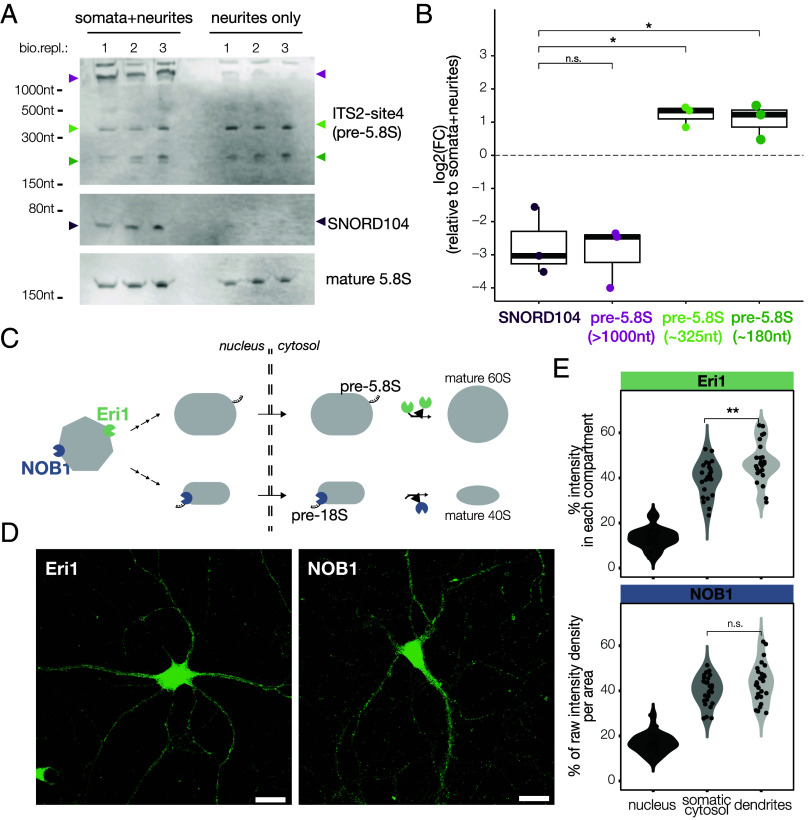
Cytosolic pre-rRNA species and their nucleases are abundant in processes. (*A*) Detection of RNA molecules of interest across subcellular compartments by Northern Blot (three biological replicates). (*B*) Boxplot of the normalized intensity levels of the indicated RNA molecules in the neurite-only compartment (data shown in *A*). Each dot represents an independent biological replicate. Anova, *P* = 0.00014; *t* test between SNORD104 and pre-5.8S (>1,000 nt), *P* = 0.78; *t* test between SNORD104 and pre-5.8S (~325 nt), *P* = 0.015; *t* test between SNORD104 and pre-5.8S (~180 nt), *P* = 0.011. (*C*) Schematic representation of the incorporation and dissociation of the endonuclease NOB1 and the exonuclease Eri1 (respectively in blue and green) during the maturation of the small and large ribosomal subunits (in gray). (*D*) Representative images showing the localization (within individual neurons) of the endogenous nucleases NOB1 and Eri1 (Scale bar, 25 μm). (*E*) Violin plot of the percentage of raw intensity density per area. Each dot represents an individual neuron, from four independent experiments. Pairwise comparisons were performed between compartments of the same individual neuron. For Eri1, Kruskal−Wallis, *P* = 2.9e−12; *t* test between somatic cytosol and dendrites, *P* = 0.0033. For NOB1, Kruskal−Wallis, *P* = 2.9e−13; *t* test between somatic cytosol and dendrites, *P* = 0.2.

To validate the sequencing results and understand better the distribution of different pre-rRNA molecules within neurites, we adapted a highly sensitive nonradioactive Northern Blot method for small RNAs (41) and used it to detect pre-5.8S molecules in neuronal compartments. We designed a 24 nt long probe conservatively close to the 3′ end of the mature 5.8S that would not only recognize 4 out of the 5 pre-5.8S molecules identified by our pre-rRNAseq, but also longer pre-rRNA species from earlier biogenesis steps. Additionally, we used a probe against the snoRNA C/D box 104 (SNORD104) as a nuclear control and a probe against the mature 5.8S rRNA as a loading control. We then used compartmentalized chambers to purify RNAs from either the somata+neurite or neurite-only compartment and processed them for Northern Blot. The pre-rRNA probe yielded a double band at around ~180 nt (corresponding to the 179 and 187 nt long species identified in our sequencing data), a second band just above 300 nt (corresponding to the 272 and 278 nt long species identified by sequencing), a third band above the 1,000 nt marker and some extra signal in the well bottom, most likely representing even larger RNA molecules which did not enter the gel (Fig. 6*A*). Based on our own data and previous studies in humans (30, 42, 43), we interpreted the 180 nt and 300 nt bands as cytosolic pre-5.8S and the >1,000 nt band as one of the last nuclear pre-5.8S species. We observed a very strong de-enrichment of the nuclear 1,000 nt long pre-5.8S in the neurite fraction, which was also observed for SNORD104, as expected (Fig. 6 *A* and *B*)—confirming the purity of our preparation. Both cytosolic pre-5.8S species were, in contrast, highly abundant in the neurite compartment, and even slightly enriched compared to the somata + neurite compartment (Fig. 6 *A* and *B*). Altogether, our data dovetail with the detection of cytosolic RBFs in dendrites and show that cytosolic (but not nuclear) pre-rRNAs are localized to neuronal processes.

### The Enzymes for Late Stages of rRNA Processing Are Present in Neuronal Processes.

Finally, we asked whether the machinery necessary to carry out the late states of rRNA processing is present in neuronal processes and whether distally localized pre-rRNA molecules could undergo local maturation. The endoribonuclease NOB1 is known to stably associate with the Internal Transcribed Spacer ITS1 in the nucleolus and to catalyze the removal of the final part of ITS1 (site D/3) in the cytosol, generating the mature 18S rRNA (44, 45) (Fig. 6*C*). The exonuclease Eri1 transiently interacts with the 5′ External Transcribed Spacer ETS in the nucleolus and catalyzes the final 3′ trimming step of the Internal Transcribed Spacer ITS2 to generate the mature 5.8S rRNA in the cytosol (46) (Fig. 6*C*). We performed immunostaining in cultured hippocampal neurons with antibodies against NOB1 and Eri1. As expected, the antibodies to both nucleases gave a strong signal in the nucleolus. The cytoplasmic signal, however, was not confined to the perinuclear region but distributed throughout the dendrites, present at either similar (NOB1) or slightly higher (Eri1) levels than observed in the perinuclear region (Fig. 6 *D* and *E*). Altogether, these data show that cytosolic pre-rRNAs are localized to dendrites where the machinery is present for their local processing.

## Discussion

Using an array of methods, including both targeted and unbiased strategies, we describe here the localization of immature ribosomes in the dendrites and/or axons of neurons. Our findings reveal that all constituent elements of cytosolic, but not nuclear, premature ribosomes, including both RNA and protein components, are present in neuronal processes. Contrary to prevailing models which assume that ribosome biogenesis concludes in the perinuclear region, with only fully assembled ribosomes being subsequently transported to distal subcellular compartments, our observations indicate a significant presence of premature ribosomal particles within neuronal processes.

We found that the abundance of immature ribosomes in neuronal processes was substantial and comparable to their levels within the cell body. This observation suggests that they are actively localized, and further studies are clearly needed to dissect transport mechanisms. RNA granules have already been shown to contain translationally incompetent ribosomes, defined by the presence of RPs and rRNAs, but the absence of transfer RNAs (tRNAs), translation factors like eIF4E and 4G as well as the inability to incorporate amino acids (17, 47). All of these features would apply also to premature ribosomes. However, additional studies are required to query the presence of RBFs or pre-rRNA species in such granules and investigate their potential trafficking via microtubules.

Additionally, neurons may localize immature ribosomes in their distal processes also via the ER. In fact, a recent publication reported a specific subpopulation of 60S ribosomes tethered to the ER of mammalian cell lines and bound by the cytosolic RBFs Lsg1, Ndm3, Zfp622, and eIF6 (48). Interestingly, we also observed these factors at high levels in neuronal processes (Fig. 2*C*). However, their potential association with ER needs to be tested.

We observed that markers of preribosomal particles from the latest steps of ribosome biogenesis tend to be particularly enriched in neuronal processes (axons and dendrites) compared to those associated with earlier steps (Fig. 3 *C*–*F*). We also observed that three RBFs (RSL24D1, Bystin, and RIOK2) were present within synaptic fractions purified from the rat cortex. One of these factors, RIOK2, was present in synapses at levels comparable to the integral synaptic scaffolding molecule, PSD-95 (Fig. 3 *E* and *F*). These data suggest that neurons could be poised to complete ribosome maturation very rapidly, for example, in response to external or synaptic stimuli. Together with other mechanisms neurons use to reorganize their translational machinery and alter protein synthesis during plasticity (3, 7, 15, 49), our data suggest the local maturation of ribosomes could efficiently boost the local capacity for translation. This potential regulation could be especially relevant in small compartments, such as the dendritic spine, where protein concentrations are tightly regulated and minor changes can have substantial effects. In all our datasets, we consistently observed that the proportion of mature rRNA and RPs is enriched in the cell body, while pre-rRNAs and RBFs tend to be equally distributed throughout the cytosol (including dendrites). Effectively, this results in a higher ratio of immature-to-mature ribosomes specifically in neuronal processes. We propose that the on-demand maturation of immature ribosomes could have a large impact near synapses, resulting in the fine-tuned regulation of the number of ribosomes able to supply a synaptic neighborhood with protein synthesis.

Finally, we note that the mRNAs encoding RPs that are incorporated during the cytosolic steps of ribosome biogenesis tend to be more abundant in dendrites compared to those of early nuclear steps (*SI Appendix,* Fig. S3*B*). We hypothesize that neurons could use the context-dependent translation of RP mRNAs as a mechanism for the rapid local activation of immature ribosomes. Additionally, via the differential incorporation of locally synthesized RPs (25), immature ribosomes might acquire different properties during the last steps of biogenesis that could differentiate them from the local pool of preexisting ribosomes. Altogether, our data suggest a potential additional mechanism for protein synthesis regulation near synapses.

## Materials and Methods

### Animals.

All animals were housed on a 12/12-h light/dark cycle with food and water ad libitum until sacrifice for tissue collection.

### Preparation of Primary Cultured Neurons.

Dissociated rat hippocampal or cortical neuron cultures were prepared and maintained as described previously (50). Briefly, we dissected hippocampi or cortices from postnatal day 0 to 2 rat pups of either sex (Sprague–Dawley strain; Charles River Laboratories) and dissociated the samples with papain (Sigma). For imaging experiments, hippocampal neurons were plated at a density of 30 × 10^3^ cells/cm^2^ (for antibody staining against endogenous proteins) or 40 × 10^3^ cells/cm^2^ (for transfection experiments) on poly-D-lysine–coated glass-bottom petri dishes (MatTek P35G-1.5-14-C). For biochemical experiments, cortical neurons were plated at a density of 4 × 10^6^ cells/cm^2^ on poly-D-lysine-coated 10 cm dishes or at a density of 9 × 10^6^ cells/cm^2^ on poly-D-lysine-coated 75 mm inserts (compartmentalized chambers; 3.0 μm pore size, NEST 726001). One day after plating on the inserts, cells were incubated with 5 μM AraC (Sigma C1768) for 2 d, then the AraC was removed by changing the media. Neurons were maintained in a humidified atmosphere at 37 °C and 5% CO_2_ in growth medium [Neurobasal-A supplemented with B27 and GlutaMAX-I (Life Technologies)] for 14 to 16 Days In Vitro (DIV) for biochemical experiments or for 25 to 27 DIV for imaging experiments.

### Immunostaining of Primary Cultured Neurons.

Cells were fixed for 20 min in 4% Paraformaldehyde in 4% sucrose in Phosphate-buffered saline (PBS), permeabilized for 15 min in 0.5% Triton-X-100+blocking buffer, and blocked for at least 30 min in blocking buffer (PBS+4% goat serum). Neurons were incubated for 2 h with primary antibodies (*SI Appendix,* Table S2) and, after three washes, for 1 h with secondary antibodies (*SI Appendix,* Table S2), all in blocking buffer. After two washes in PBS, cells were stained with DAPI (1:1,000 for 2 min) and kept in PBS at 4 °C until imaging. For validation of the compartmentalized chambers (Fig. 1*A*), pieces of the filter were processed as described above and mounted on a glass slide (ThermoFisher 10417002) with Aqua Poly/mount (Polysciences, 18606). Samples were imaged using LSM780 confocal microscopy (Zeiss) using a Plan-Apochromat 40×/1.4 Oil Differential interference contrast (DIC) M27 or Plan-Apochromat 20×/0.8 M27 objectives. A z-stack was set to cover the entire volume of the neuron, with optical slice thickness set to optimal and number of slices kept equal across conditions of the same experiment. Laser power and detector gain were adjusted to avoid saturated pixels. Imaging conditions were held constant within experiments. Average intensity projections of z-stacks were used for image analysis. For visualization purposes (but not analyses) brightness and contrast were adjusted.

### Image Analysis.

All image analyses were performed in ImageJ/FIJI (Version 2.1.0/1.53c) with a semiautomated script built in-house. Briefly, the soma and dendritic arbor of individual neurons were manually traced based on the MAP2-positive signal. The nucleus was automatically determined using the DAPI signal. The somatic cytosol area was determined by subtracting the nuclear (DAPI-positive) area from the manually traced somatic area. The raw intensity of the channel of interest (the Dendra2-fluorescent reporter for Fig. 3 *B*–*D* or the antibody staining for Fig. 6 *D* and *E*) was then measured in each area. Additionally, for Fig. 2 *D* and *E*, the background intensity in empty regions was subtracted from the intensity in the neuronal subcellular compartment from the same field of view. Statistical testing and plotting were then performed in RStudio (Version 2023.06.1+524).

### Sample Collection from Compartmentalized Chambers for Protein analysis.

Both sides of the inserts were washed three times with ice-cold Rnase-free Dulbecco's Phosphate-Buffered Saline (DPBS) (ThermoFisher, 14040-091) supplemented with 100 µg/mL of Cycloheximide (CHX) (Sigma, C7698). The upper compartment (containing somata and neurites) was scraped in 400 µL of RNAse-free PBS. The lower compartment (containing neurites only) was scraped twice in 100 µL of ribosome lysis buffer (20 mM Tris pH 7.4, 150 mM NaCl, 5 mM MgCl2, 24 U/mL TurboDNase, 100 µg/mL cycloheximide, 1% TritonX-100, 1 mM Dithiothreitol (DTT), RNasin(R) Plus RNase inhibitor 200 U/mL, and 1× cOmplete Ethylenediaminetetraacetic acid (EDTA)-free protease inhibitor). To guarantee sufficient yield from the neurite compartment, each biological replicate consisted of the content of two inserts pooled together. Each sample was then split (10% used for total lysate, 90% for sucrose cushioning). The samples from the upper compartment were centrifuged at 500× g at 4 °C for 5 min, supernatant was discarded, and cell pellets were used for downstream processing.

### Sample Collection from Compartmentalized Chambers for RNA Analysis.

Both sides of the inserts were washed three times with ice-cold Rnase-free PBS. The upper compartment (containing cell body and neurites) was scraped in 400 µL of RNAse-free PBS. The lower compartment (containing neurites only) was scraped twice in 100 µL of RNAse-free PBS. All samples were centrifuged for 5 min at 500× g at 4 °C. Most of supernatant was discarded, leaving only 200 µL of volume. 600 µL of Trizol LS solution (ThermoFisher, 10296010) was added to each sample and mixed well by vortexing. Samples were either immediately used for RNA purification or stored at −80 °C. Samples containing soma+neurites were purified using Direct-zol RNA Miniprep Plus (Zymo Research, R2072) and eluted in 50 µL of Rnase-free water. Samples containing neurites only were purified using Direct-zol RNA Microprep (Zymo Research, R2062) and eluted in 10 µL of Rnase-free water. In both cases, the manufacturer instructions were followed (including the DNAse I step). The RNA content from two inserts was pooled to have enough material for downstream experiments.

### Total Cell Lysates.

Cells were lysed in 200 µL (upper compartment) or 50 µL (lower compartment) of 8 M urea, 200 mM Tris/HCl [pH 8.4], 4% 3-((3-cholamidopropyl) dimethylammonio)-1-propanesulfonate) (CHAPS), 1 M NaCl, cOmplete EDTA-free protease inhibitor (Roche, 11873580001), using a pestle. Lysates were sonicated at 4 °C for four rounds of 30 s each and incubated for 10 min with 1 µL of Benzonase (Sigma E1014). After centrifugation for 5 min at 10,000× g, the supernatant was saved, and protein concentration was measured with the BCA assay (ThermoFisher, 23252). Samples (between 10 to 60 µg) were then submitted to MS.

### Sucrose Cushion.

Cell pellets from the upper compartments were resuspended in 400 µL of ribosome lysis buffer. All samples were pipetted up and down with a 0.4 × 20mm syringe needle (HSW FINE-JECT) on ice until homogenization was clear. Samples were then centrifuged at 10,000× g for 10 min at 4 °C. Supernatants were loaded on 1 mL sucrose solution (34% sucrose, 20 mM Tris pH 7.4, 150 mM NaCl, 5 mM MgCl_2_, 1 mM DTT, 100 µg/mL cycloheximide) in a thickwall polycarbonate tube (Beckman, 349622) and centrifuged for 3 h and 30 min at 4 °C at 55,000 rpm (367,600× g) with a SW55Ti rotor (acceleration 0, deceleration 7). Ribosome pellet was resuspended in 30 µL of 10 mM N-2-Hydroxyethylpiperazine-N-2-ethanesulfonic acid (HEPES), 120 mM NaCl, 3 mM KCl, 10 mM D-Glucose, 2 mM MgSO_4_, and 2 mM CaCl_2_ and submitted to MS.

### Western Blot Validation of Sucrose Cushioning.

Cells from two 10 cm dishes were washed three times with 10 mL of ice-cold Rnase-free DPBS (ThermoFisher, 14040-091) and then scraped in 500 µL of ice-cold Rnase-free DPBS supplemented with 100 μg/mL cycloheximide. Cells were then pelleted at 500× g for 5 min at 4 °C and the supernatant discarded. Cell pellets were then resuspended in 500 μL ribosome lysis buffer. All samples were pipetted up and down until homogenization was clear with a 0.4 × 20 mm syringe needle (HSW FINE-JECT) on ice. Samples were then centrifuged at 10,000× g for 10 min at 4 °C. 200 µL of the supernatants (cleared lysates) were loaded on 1 mL sucrose solution in a thickwall polycarbonate tube (Beckman, 349622) and centrifuged for 30 min or for 3 h and 30 min at 4 °C at 55,000 rpm (367600× g) with a SW55Ti rotor (acceleration 0, deceleration 7). Ribosome pellet was resuspended in 20 μL of 10 mM HEPES, 120 mM NaCl, 3 mM KCl, 10 mM D-Glucose, 2 mM MgSO_4_, and 2 mM CaCl_2_ and submitted to western blot. 6 µL of the clear lysates (input, 3% of the input total volume) or 10 µL of the cushion samples (50% of the total volume) were mixed with NuPAGE LDS Sample Buffer (ThermoFisher, NP007) and NuPAGE Sample Reducing Agent (ThermoFisher, NP004) to a final concentration of 1×, and then loaded onto a 4 to 12% Bis-Tris NuPAGE gel (ThermoFisher). Gels were transferred using the Trans-Blot Turbo Transfer Pack (Biorad, 1704157) on a polyvinylidene fluoride membrane (Immobilon-FL, IPFL00010 0.45 μm pore size). Membranes were stained using Revert 700 Total Protein Stain (LI-COR, 926-11015) to access loading, and then destained according to the manufacturer’s instructions. Immunoblotting was performed with primary and secondary antibodies (*SI Appendix,* Table S2), diluted in Intercept Blocking Buffer (LI-COR 927-70001). 0.1% Tween in PBS was used for washes. Images were acquired using LI-COR Image Studio Lite (version 3.0.30, RRID:SCR_013715). Band intensity was quantified with AzureSpot Pro (v1.0 – 366, Azure Biosystems). Image analysis was then performed in RStudio (Version 2023.06.1+524).

### Synaptosome Preparation.

For each replicate, one Cortex hemisphere dissected from 8 to 9 wk old male Sprague Dawley rats (CD, Charles River Laboratories) after decapitation under deep isoflurane anesthesia was homogenized in a Glass-Douncer in 5 mL SynPer reagent (ThermoFisher) supplemented with Protease Inhibitor Cocktail III (Calbiochem) by 10 careful strokes with a loose and 10 strokes with a tight pestle on ice. An aliquot of the homogenate was set aside, and 4 mL of the homogenate was centrifuged three times for 10 min at 4 °C at 1,200× g to ensure efficient removal of cell debris and nuclei. Subsequently, the Synaptoneurosomes (SN) were collected as pellet from 3 mL of the supernatant by centrifugation for 20 min at 15,000× g at 4 °C and resuspended in 500 µL SynPer reagent with protease inhibitor. Lysates were prepared from homogenate and SN fractions by addition of water, 4× LDS sample buffer (ThermoFisher), and 10× reducing agent (ThermoFisher) to a final concentration of 1 μg/μL total protein, and 20 μg was separated on NuPAGE 4 to 12% BIS TRIS 1.5 mm gels (ThermoFisher) with 3-(N-morpholino)propanesulfonic acid (MOPS) running buffer and blotted with the Trans-Blot Turbo Transfer system (BioRAD) onto Nitrocellulose membranes. Total protein was visualized by staining with Ponceau S solution (ThermoFisher). Primary antibodies were applied overnight at 4 °C, IRDye680-coupled secondary antibodies against the respective species were used 1:5,000 and applied for 1 h at room temperature prior to scanning on an Azure Sapphire imager.

### MS Sample Preparation and Analysis Summary.

Proteins were digested using the S-Trap protocol, and peptides were desalted, dried, and stored at −20 °C until Liquid Chromatography-Mass Spectrometry (LC–MS) analysis. Peptides were separated via nano-High-Performance Liquid Chromatography (HPLC) on C18 columns and analyzed using a Fusion Lumos mass spectrometer in DIA mode with a 120-min gradient. DIA raw files were processed using DIA-NN in a library-free mode with in silico digestion and deep learning-based spectrum prediction. For further details, please refer to *SI Appendix, Supplementary Methods*.

### Molecular Cloning.

Constructs containing Dendra2 fused to a Ribosome Biogenesis Factor (Pescadillo, DIMT1, or RIOK2) or Ribosomal Protein RPS14 were cloned using Gibson Assembly (NEB, E5510) and standard cloning techniques. In each case, the insert for Dendra2 was obtained as a gBlocks Gene Fragment (IDT), and the CAG vector backbone was derived from CRY2-GFP-homer1c (Addgene plasmid #89442, a gift from Matthew Kennedy, University of Colorado School of Medicine, AUR) by XhoI and EcoRV digestion to excise CRY2-GFP-homer1c. The unique inserts for the DIMT1 and RIOK2 constructs were amplified by PCR from DIMT1_pLX307 (Addgene plasmid #98330, a gift from William Hahn & Sefi Rosenbluh, Broad Institute of Harvard and Massachusetts Institute of Technology, MA) and pDONR223-RIOK2 (Addgene plasmid #23535, a gift from William Hahn & David Root, Broad Institute of Harvard and Massachusetts Institute of Technology, MA), respectively. The unique insert for RPS14 (sequence provided in *SI Appendix,* Table S3) was amplified by PCR from a pcDNA3.1 vector (Invitrogen, V79020) containing the RPS14 open reading frame. The unique insert for Pescadillo was obtained as a gBlocks Gene Fragment (IDT).

### Northern Blot.

Total RNA (1 µg) was denatured and resolved on 10% Tris-borate-EDTA (TBE)-Urea gels, stained with SYBR Gold, and transferred to a nylon membrane using semidry electroblotting. RNA was crosslinked using 1-Ethyl-3-(3-dimethylaminopropyl)carbodiimide hydrochloride (EDC) chemistry. DIG-labeled probes targeting ITS2-site4, SNORD104, and mature 5.8S rRNA were synthesized using terminal transferase and DIG-dUTP. Hybridization was performed overnight or for 30 min depending on the probe, followed by stringent washes. DIG detection was achieved using anti-DIG-AP antibody and CSPD chemiluminescent substrate. Imaging was done on an Azure system, and band intensities were quantified with AzureSpot Pro and further analyzed in R. Membranes were stripped and reused for additional probes. For further details, please refer to *SI Appendix, Supplementary Methods*.

### Gel Electrophoresis for Pre-rRNA Sequencing.

Purified RNA samples were mixed with an equal volume of 2× Novex TBE-Urea Sample Buffer (ThermoFisher, LC6876). Samples were denatured at 80 °C for 90 s and returned to ice before being loaded on a denaturing Novex 10% TBE-Urea Gels (ThermoFisher, EC6875BOX) in 1× TBE running buffer at 200 V for 50 min. Total RNA was visualized with SYBR Gold Nucleic Acid Gel Stain (ThermoFisher, S11494). Gel bands in the range of 160 nt to 400 nt were excised and crushed with disposable RNAse-free pestles (FisherScientific, 13236679). The crushed gels had the addition of 100 µL gel elution buffer composed of 0.3 M sodium acetate pH = 4.5, 0.25% sodium dodecyl sulfate (SDS), 1 mM EDTA pH = 8.0, as previously described (51). RNA was eluted for 10 min at 65 °C and shaking at 1,400 rpm in a thermal mixer. Gel pieces were removed with Spin-X centrifuge tube filter 0.22 µm (Costar, Corning 8169). RNA was purified by using the RNA Clean & Concentrator-5 Kit (Zymo Research, R1013) and eluted in 6 µL of RNase-free water. The yield of RNA was quantified using Qubit HS-RNA (ThermoFisher, Q32855). Samples were either processed immediately for library prep or stored at −80 °C.

### RNA Library Prep.

For end repair, 30 ng of RNA was mixed with 0.2 U/µL T4 PNK (NEB, M0201), 1.2 U/µL SUPERase inhibitors (ThermoFisher, AM2694) in 2× T4 RNA Ligase Buffer (NEB, B0216L), and incubated at 37 °C. for 30 min and at 65 °C for 20 min, before returning to ice. For 3′ adapter ligation, samples were then incubated at room temperature for 3 h under constant motion, in 1 µM Adenylated Fusion Oligo (Table below), 10 U/µL Truncated KQ Rnl2 (NEB, M0373) in 25% PEG 8000. Unligated adapters were digested by adding 2.2 µL of a mixture of equal volume of Deadenylase (NEB, M0331S) and Exonuclease VIII (NEB, M0545S) to the samples at 30 °C for 15 min for deadenylation, followed by 37 °C for 30 min for digestion, and then at 75 °C for 10 min to inactivate the enzymes. For RT Primer Activation, samples were mixed with 0.25 U/µL RNAse HII (NEB, M0288) in CutSmart buffer (NEB, B7204) and incubated at 37 °C for 30 min, and then at 75 °C for 10 min to inactivate the enzymes. RT was performed by incubating samples at 42 °C for 16 h, with 10 U/µL Induro RT (NEB, M0681), 0.5 U/µL SUPERase inhibitors (ThermoFisher, AM2694), 1 mM deoxynucleoside triphosphate (dNTPs), 5 mM DTT in 1× NEBNext First-strand buffer (NEB, E7421AVIAL). To digest and inactive the reverse transcriptase, samples were incubated with 16 U/µL Proteinase K (NEB, P8107S) solution at 25 °C for 10 min and then at 75 °C for 5 min. To digest RNA, samples were incubated with 0.11 U/µL RNase H (NEB, M0297) and 1.1 U/µL RNase If (NEB, M0243) at 37 °C for 30 min and then at 70 °C for 30 min. Unligated adapters were digested by adding 1 U/µL Exonuclease III (NEB, M0206) in CutSmart buffer (NEB, B7204) to the samples at 30 °C for 15 min for deadenylation, followed by 37 °C for 30 min for digestion, and then at 70 °C for 30 min to inactivate the enzymes. cDNA polyadenylation was performed by adding 2 U/µL Terminal Transferase (NEB, M0315), 3 mM deoxyadenosine triphosphate (dATP) and incubating the samples at 37 °C for 1 min and at 75 °C for 20 min. Uracil DNA digestion was performed by adding 0.02 U/µL Thermolabile USER II (NEB, M5508S), 0.1 U/µL rSAP (NEB, M0371), 1 µM 2nd Strand_OligoT (Table below), and incubating the samples at 37 °C for 30 min and at 65 °C for 10 min. The second strand was synthesized by incubating samples with 1 U/µL Large Klenow Fragment DNA Pol (NEB, M0210M), 2 mM dNTP mix at 25 °C for 60 min and at 75 °C for 20 min. Samples were stored at 4 °C overnight. cDNA amplification was performed by adding 1 µM small RNA (smRNA) PCR1 universal primer and 1 µM smRNA PCR_iX index primer (Step III) (Table below) and 1× KAPA HiFi HotStart Ready Mix (Roche, 09420398001) to the ⅓ (30 µL) of 2nd strand synthesis reaction. Initial denaturation occurred at 98 °C for 30 s, followed by 19 cycles consisting of a denaturation step at 98 °C for 5 s, an annealing step at 60 °C for 10 s, and an extension step at 72 °C for 30 s. This was followed by a final extension at 72 °C for 5 min.

**Table:** 

Primer or oligo name	Sequence
Adenylated Fusion Oligo	5Phos/GTANNNNNNNNAGCTAGAUCGTCGGACTGUAGAACTCTUCTATGGAGCCTGTTCAGAGTTCTACAGTCCGrACGAT/3ddC/
2ndStrand_OligoT	AGACGTGTGCTCTTCCGATCTTTTTTTTTTTTTTTTTT
smRNA PCR1	AATGATACGGCGACCACCGAGATCTACACGTTCAGAGTTCTACAGTCCG
smRNA PCR_iX	CAAGCAGAAGACGGCATACGAGATNNNNNNGTGACTGGAGTTCAGACGTGTGCTCTTCCGATCT)

PCR products were purified with Magnetic DNA purification beads (AMPure XP, Beckman Coulter, A63881) according to the manufacturer’s instructions and eluted in 23 µL Buffer EB (Qiagen, 19086). DNA samples were mixed with 6× Gel loading dye (NEB, B7024S) and ran on a 20% TBE gel at 100 V for 5 h. Gels were visualized with SYBR Gold Nucleic Acid Gel Stain (ThermoFisher, S11494). Gel bands in the range of 200 nt to 300 nt were excised and crushed with disposable RNAse-free pestles. T 20µL of water was added to the crushed gels before being incubated at 4 °C for at least 10 h. Gel pieces were removed with Spin-X centrifuge tube filters (Costar, 8169), and DNA was measured using the Qubit dsDNA HS kit (ThermoFisher, Q32854) and the HS DNA Bioanalyzer Assay (Agilent, 5067).

Sequencing was performed using the Illumina NextSeq 2000 system, operating in single-end read mode. This involved a P5 read1 cDNA sequence over 100 cycles and an I7 sample index over six cycles, both in the forward direction. Read1 commenced with a 5 bp index, an 8 bp Unique Molecular Identifier (UMI) followed, and a 3 base anchor (ATG).

### Analysis.

For data demultiplexing, we utilized bcl2fastq with a unique base mask (--use-base-mask N5Y*,I6) to exclude the 5 bp integrated index from the cDNA’s 5′end. The reads underwent cleaning to remove adapters and low-quality segments using [fastp](https://github.com/OpenGene/fastp). In this process, the UMI was trimmed and added to the header, and the three anchor bases were disregarded. The resulting clean reads began with either 3 or 2 base charging states.

Our analysis included preparing a Rat transcriptome reference using the latest rn7 annotation, incorporating the labeling format: <transcript_id>|<gene_symbol>|<rna_type>. Reads were aligned using [bowtie2] (https://github.com/BenLangmead/bowtie2), reducing the local alignment’s anchor size to 15. This adjustment was based on the premise that modifications should be detectable within a 15-base window. The alignment parameters were set as follows: “-D 20 -R 3 -N 0 -L 15 -i S,1,0.50.”

For gene count tabulation, we developed a custom script. The DESeq2 package facilitated quality control through PCA plots and differential expression testing., All pipeline code is accessible in the [trnatools] (https://gitlab.mpcdf.mpg.de/mpibr/schu/trnatools) repository.

## Supplementary Material

Appendix 01 (PDF)

Dataset S01 (XLSX)

Dataset S02 (XLSX)

Dataset S03 (XLSX)

Dataset S04 (XLSX)

Dataset S05 (XLSX)

Dataset S06 (XLSX)

## Data Availability

The mass spectrometry proteomics data have been deposited to the ProteomeXchange Consortium via the PRIDE partner repository with the dataset identifier PXD058169. The pipeline code used for pre-rRNAseq data is accessible in the trnatools (https://gitlab.mpcdf.mpg.de/mpibr/schu/trnatools) repository. All data are included in the article and/or supporting information.

## References

[1] D. Bodian, A suggestive relationship of nerve cell RNA with specific synaptic sites. Proc. Natl. Acad. Sci. U.S.A. **53**, 418–425 (1965). 14294076 10.1073/pnas.53.2.418 PMC219529

[2] L. E. Ostroff, J. C. Fiala, B. Allwardt, K. M. Harris, Polyribosomes redistribute from dendritic shafts into spines with enlarged synapses during LTP in developing rat hippocampal slices. Neuron **35**, 535–545 (2002). 12165474 10.1016/s0896-6273(02)00785-7

[3] C. Sun , The prevalence and specificity of local protein synthesis during neuronal synaptic plasticity. Sci. Adv. **7**, eabj0790 (2021). 34533986 10.1126/sciadv.abj0790 PMC8448450

[4] V. M. Tennyson, The fine structure of the axon and growth cone of the dorsal root neuroblast of the rabbit embryo. J. Cell Biol. **44**, 62–79 (1970). 5409464 10.1083/jcb.44.1.62 PMC2107779

[5] A.-S. Hafner, P. G. Donlin-Asp, B. Leitch, E. Herzog, E. M. Schuman, Local protein synthesis is a ubiquitous feature of neuronal pre- and postsynaptic compartments. Science **364**, eaau3644 (2019). 31097639 10.1126/science.aau3644

[6] A. M. Bourke, A. Schwarz, E. M. Schuman, De-centralizing the central dogma: MRNA translation in space and time. Mol. Cell **83**, 452–468 (2023). 36669490 10.1016/j.molcel.2022.12.030

[7] C. E. Holt, K. C. Martin, E. M. Schuman, Local translation in neurons: Visualization and function. Nat. Struct. Mol. Biol. **26**, 557–566 (2019). 31270476 10.1038/s41594-019-0263-5

[8] M. Costa-Mattioli, W. S. Sossin, E. Klann, N. Sonenberg, Translational control of long-lasting synaptic plasticity and memory. Neuron **61**, 10–26 (2009). 19146809 10.1016/j.neuron.2008.10.055 PMC5154738

[9] S. Das, M. Vera, V. Gandin, R. H. Singer, E. Tutucci, Intracellular mRNA transport and localized translation. Nat. Rev. Mol. Cell Biol. **22**, 483–504 (2021). 33837370 10.1038/s41580-021-00356-8 PMC9346928

[10] G. V. D. Prisco , Translational control of mGluR-dependent long-term depression and object-place learning by eIF2α. Nat. Neurosci. **17**, 1073–1082 (2014). 24974795 10.1038/nn.3754 PMC4340591

[11] Y. Shen , PQBP1 promotes translational elongation and regulates hippocampal mGluR-LTD by suppressing eEF2 phosphorylation. Mol. Cell **81**, 1425–1438.e10 (2021). 33662272 10.1016/j.molcel.2021.01.032

[12] C. M. Fusco, “Ribosomal protein dynamics and plasticity in the cell body and processes of neurons,” Dissertation thesis, Johann Wolfgang Goethe-Universitaet Frankfurt am Main, Germany (2024).

[13] J. Bohlen, M. Roiuk, A. A. Teleman, Phosphorylation of ribosomal protein S6 differentially affects mRNA translation based on orf length. Nucleic Acids Res. **49**, 13062–13074 (2021). 34871442 10.1093/nar/gkab1157 PMC8682771

[14] Z. A. Knight , Molecular profiling of activated neurons by phosphorylated ribosome capture. Cell **151**, 1126–1137 (2012). 23178128 10.1016/j.cell.2012.10.039 PMC3839252

[15] S. S. Seo , Excess ribosomal protein production unbalances translation in a model of Fragile X syndrome. Nat. Commun. **13**, 3236 (2022). 35688821 10.1038/s41467-022-30979-0 PMC9187743

[16] J.-M. Cioni , Late endosomes act as mRNA translation platforms and sustain mitochondria in axons. Cell **176**, 56–72.e15 (2019). 30612743 10.1016/j.cell.2018.11.030 PMC6333918

[17] A. M. Krichevsky, K. S. Kosik, Neuronal RNA granules a link between RNA localization and stimulation-dependent translation. Neuron **32**, 683–696 (2001). 11719208 10.1016/s0896-6273(01)00508-6

[18] I. J. Cajigas , The local transcriptome in the synaptic neuropil revealed by deep sequencing and high-resolution imaging. Neuron **74**, 453–466 (2012). 22578497 10.1016/j.neuron.2012.02.036 PMC3627340

[19] A. Biever , Monosomes actively translate synaptic mRNAs in neuronal processes. Science **367**, eaay4991 (2020). 32001627 10.1126/science.aay4991

[20] J. D. Perez , Subcellular sequencing of single neurons reveals the dendritic transcriptome of GABAergic interneurons. Elife **10**, e63092 (2021). 33404500 10.7554/eLife.63092 PMC7819707

[21] J. D. Perez, C. M. Fusco, E. M. Schuman, A functional dissection of the mRNA and locally synthesized protein population in neuronal dendrites and axons. Annu. Rev. Genet. **55**, 1–25 (2021). 34460296 10.1146/annurev-genet-030321-054851

[22] R. Moccia , An unbiased cDNA library prepared from isolated Aplysia sensory neuron processes is enriched for cytoskeletal and translational mRNAs. J. Neurosci. **23**, 9409–9417 (2003). 14561869 10.1523/JNEUROSCI.23-28-09409.2003 PMC6740582

[23] A. Poulopoulos , Subcellular transcriptomes and proteomes of developing axon projections in the cerebral cortex. Nature **565**, 356–360 (2019). 30626971 10.1038/s41586-018-0847-y PMC6484835

[24] T. Shigeoka , Dynamic axonal translation in developing and mature visual circuits. Cell **166**, 181–192 (2016). 27321671 10.1016/j.cell.2016.05.029 PMC4930487

[25] C. M. Fusco , Neuronal ribosomes exhibit dynamic and context-dependent exchange of ribosomal proteins. Nat. Commun. **12**, 6127 (2021). 34675203 10.1038/s41467-021-26365-x PMC8531293

[26] T. Shigeoka , On-site ribosome remodeling by locally synthesized ribosomal proteins in axons. Cell Rep. **29**, 3605–3619.e10 (2019). 31825839 10.1016/j.celrep.2019.11.025 PMC6915326

[27] Y.-M. Yang, K. Karbstein, The chaperone Tsr2 regulates Rps26 release and reincorporation from mature ribosomes to enable a reversible, ribosome-mediated response to stress. Sci. Adv. **8**, eabl4386 (2022). 35213229 10.1126/sciadv.abl4386 PMC8880767

[28] Y.-M. Yang , Chaperone-directed ribosome repair after oxidative damage. Mol. Cell **83**, 1527–1537.e5 (2023). 37086725 10.1016/j.molcel.2023.03.030 PMC10164075

[29] M. B. Ferretti, H. Ghalei, E. A. Ward, E. L. Potts, K. Karbstein, Rps26 directs mRNA-specific translation by recognition of Kozak sequence elements. Nat. Struct. Mol. Biol. **24**, 700–707 (2017). 28759050 10.1038/nsmb.3442 PMC5777333

[30] S.-T. Mullineux, D. L. J. Lafontaine, Mapping the cleavage sites on mammalian pre-rRNAs: Where do we stand? Biochimie **94**, 1521–1532 (2012). 22342225 10.1016/j.biochi.2012.02.001

[31] D. Kressler, E. Hurt, J. Baßler, A puzzle of life: Crafting ribosomal subunits. Trends Biochem. Sci. **42**, 1–15 (2017). 28579196 10.1016/j.tibs.2017.05.005

[32] K. Dörner, C. Ruggeri, I. Zemp, U. Kutay, Ribosome biogenesis factors—From names to functions. EMBO J. **42**, e112699 (2023). 36762427 10.15252/embj.2022112699 PMC10068337

[33] K. Karbstein, Quality control mechanisms during ribosome maturation. Trends Cell Biol. **23**, 242–250 (2013). 23375955 10.1016/j.tcb.2013.01.004 PMC3640646

[34] E. L. Cerezo , RIOK2 phosphorylation by RSK promotes synthesis of the human small ribosomal subunit. PLoS Genet. **17**, e1009583 (2021). 34125833 10.1371/journal.pgen.1009583 PMC8224940

[35] E. C. Holland, N. Sonenberg, P. P. Pandolfi, G. Thomas, Signaling control of mRNA translation in cancer pathogenesis. Oncogene **23**, 3138–3144 (2004). 15094763 10.1038/sj.onc.1207590

[36] V. Iadevaia, Z. Zhang, E. Jan, C. G. Proud, mTOR signaling regulates the processing of pre-rRNA in human cells. Nucleic Acids Res. **40**, 2527–2539 (2012). 22121221 10.1093/nar/gkr1040 PMC3315323

[37] M. Prattes, Y.-H. Lo, H. Bergler, R. E. Stanley, Shaping the nascent ribosome: AAA-ATPases in eukaryotic ribosome biogenesis. Biomolecules **9**, 715–734 (2019). 31703473 10.3390/biom9110715 PMC6920918

[38] P. Nerurkar , “Eukaryotic ribosome assembly and nuclear export” in *International Review of Cell and Molecular Biology*, K. W. Jeon, Ed. (Elsevier, 2015), vol. 19, pp. 107–140. 10.1016/bs.ircmb.2015.07.002 26404467

[39] M. Ameismeier, J. Cheng, O. Berninghausen, R. Beckmann, Visualizing late states of human 40S ribosomal subunit maturation. Nature **558**, 249–253 (2018). 29875412 10.1038/s41586-018-0193-0

[40] J. de la Cruz, K. Karbstein, J. L. Woolford, Functions of ribosomal proteins in assembly of eukaryotic ribosomes in vivo. Annu. Rev. Biochem. **84**, 93–129 (2015). 25706898 10.1146/annurev-biochem-060614-033917 PMC4772166

[41] S. W. Kim , A sensitive non-radioactive northern blot method to detect small RNAs. Nucleic Acids Res. **38**, e98 (2010). 20081203 10.1093/nar/gkp1235 PMC2853138

[42] J. E. Farrar , Abnormalities of the large ribosomal subunit protein, Rpl35a, in Diamond-Blackfan anemia. Blood **112**, 1582–1592 (2008). 18535205 10.1182/blood-2008-02-140012 PMC2518874

[43] S. Schillewaert, L. Wacheul, F. Lhomme, D. L. J. Lafontaine, The evolutionarily conserved protein LAS1 is required for pre-rRNA processing at both ends of ITS2. Mol. Cell. Biol. **32**, 430–444 (2012). 22083961 10.1128/MCB.06019-11 PMC3255765

[44] A. C. Lamanna, K. Karbstein, Nob1 binds the single-stranded cleavage site D at the 3′-end of 18S rRNA with its PIN domain. Proc. Natl. Acad. Sci. U.S.A. **106**, 14259–14264 (2009). 19706509 10.1073/pnas.0905403106 PMC2732849

[45] K. E. Sloan , Both endonucleolytic and exonucleolytic cleavage mediate ITS1 removal during human ribosomal RNA processing. J. Cell Biol. **200**, 577–588 (2013). 23439679 10.1083/jcb.201207131 PMC3587827

[46] K. M. Ansel , Mouse Eri1 interacts with the ribosome and catalyzes 5.8S rRNA processing. Nat. Struct. Mol. Biol. **15**, 523–530 (2008). 18438418 10.1038/nsmb.1417 PMC3032500

[47] K. E. Bauer, B. R. Queiroz, M. A. Kiebler, F. Besse, RNA granules in neuronal plasticity and disease. Trends Neurosci. **46**, 525–538 (2023). 37202301 10.1016/j.tins.2023.04.004

[48] Z. Zhang , A subcellular map of translational machinery composition and regulation at the single-molecule level. Science **387**, eadn2623 (2025). 40048539 10.1126/science.adn2623 PMC13102253

[49] M. Koppers , Receptor-specific interactome as a hub for rapid cue-induced selective translation in axons. Elife **8**, e48718 (2019). 31746735 10.7554/eLife.48718 PMC6894925

[50] K. Desch, E. M. Schuman, J. D. Langer, Quantifying phosphorylation dynamics in primary neuronal cultures using LC-MS/MS. STAR Protoc. **3**, 101063 (2022). 35005645 10.1016/j.xpro.2021.101063 PMC8715330

[51] A. Behrens, G. Rodschinka, D. D. Nedialkova, High-resolution quantitative profiling of tRNA abundance and modification status in eukaryotes by mim-tRNAseq. Mol. Cell **81**, 1802–1815.e7 (2021). 33581077 10.1016/j.molcel.2021.01.028 PMC8062790

