# Supplementary material for: Neuronal processes contain the essential components for the late steps of ribosome biogenesis
Source: Proc Natl Acad Sci U S A. 2025 Jul 31;122(31):e2502424122. doi: 10.1073/pnas.2502424122 (PMC12337303; doi:10.1073/pnas.2502424122)
Supplement: Supplementary file 1 — Appendix 01 (PDF) [file pnas.2502424122.sapp.pdf]

Supplementary information for

Neuronal processes contain the essential components for the late steps of ribosome biogenesis

Claudia M. Fusco[1,3], Anja Staab[1], Ashley M. Bourke[1], Georgi Tushev[1], Kristina Desch[1], Erico Moreto Lins[1], Elena Ciirdaeva[1], Susanne tom Dieck[1], Nina Kaltenschnee[2], Alexander Heckel[2], Julian D. Langer[1], Erin M. Schuman[1]*

* to whom correspondence should be addressed
Email: erin.schuman@brain.mpg.de

Supplementary Tables:

- Dataset S1: manually curated list of translation-related genes (used as input to Fig. 1e)
- Dataset 2: Protein Intensity values of Ribosome Biogenesis Factors identified in total lysates across neuronal subcellular compartments (related to Table 1)
- Dataset 3: Protein Intensity values of all detected proteins in the total lysates and sucrose cushion across neuronal subcellular compartments

Source Data:

- Source Data for Figure 1: Differential expression analysis between compartments (total lysate).
- Source Data for Figure 4: Differential expression analysis between total lysate and sucrose cushion from soma+neurites compartment.
- Source Data for Suppl. Figure 3b: RP mRNA abundance per 10μm^2 of dendrite (as measured in Fusco et al. 2021) according to the order and site of RP incorporation during ribosome biogenesis.

This PDF includes:

- **Supplementary Figures:**
  - Suppl. Figure 1. Proteomic characterization of neuronal subcellular compartments.
  - Suppl. Figure 2. Proteomic characterization of sucrose cushioning from different neuronal subcellular compartments.
  - Suppl. Figure 3. Customized pre-rRNA-seq pipeline and RP mRNAs localization in dendrites.
- **Supplementary Tables:**
  - Supplementary Table 1. Manually curated list of Ribosome Biogenesis Factors.
  - Supplementary Table 2. List of antibodies.
  - Supplementary Table 3. Sequence of Primers and gBlocks used for molecular cloning.
- **Supplementary Methods:**
  - Mass Spectrometry sample preparation, acquisition and analysis.
  - Northern Blot sample preparation, acquisition and analysis.

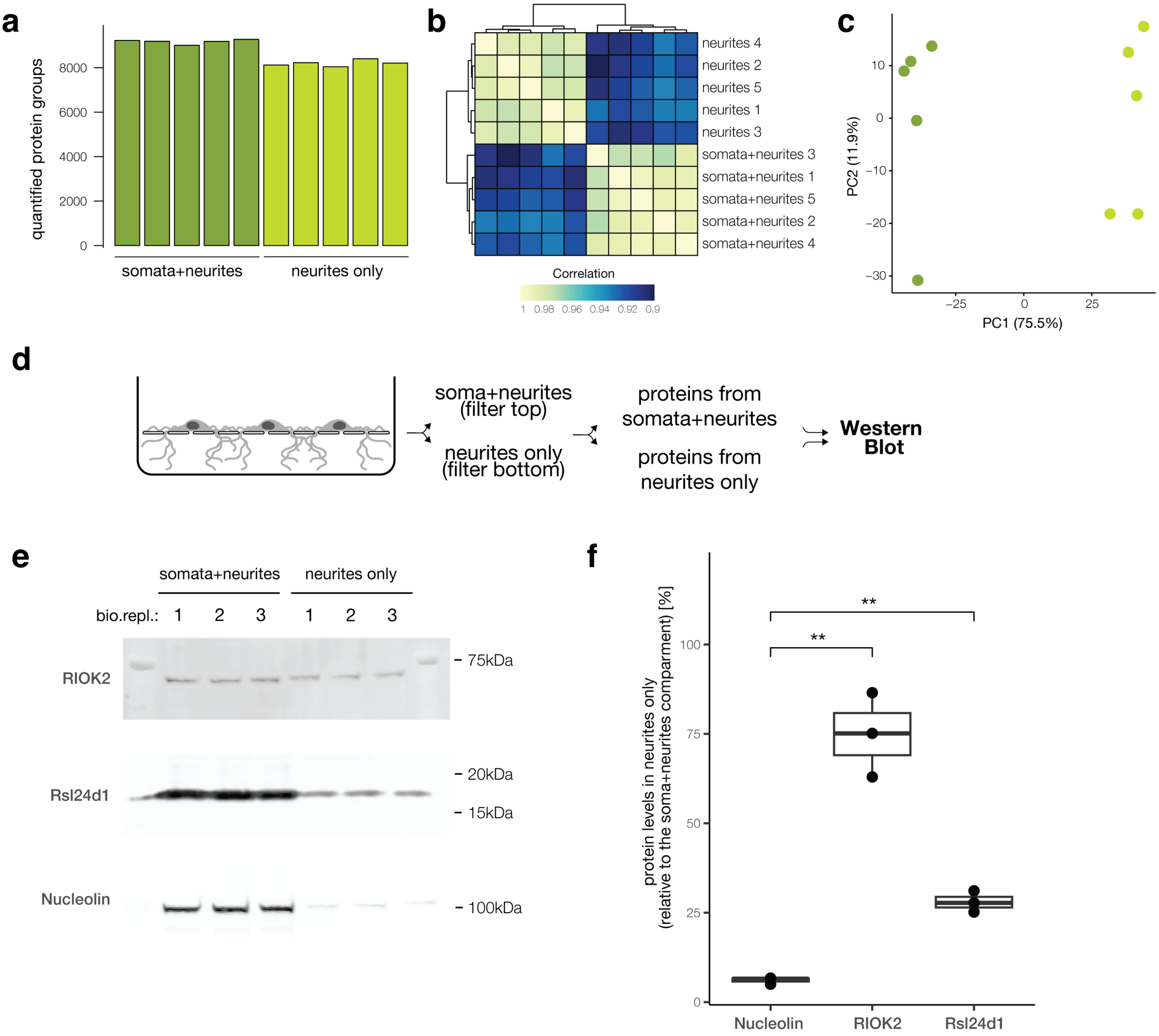
a
quantified protein groups
8000
6000
4000
2000
0
somata+neurites
neurites only
b
neurites 4
neurites 2
neurites 5
neurites 1
neurites 3
somata+neurites 3
somata+neurites 1
somata+neurites 5
somata+neurites 2
somata+neurites 4
Correlation
1 0.98 0.96 0.94 0.92 0.9
c
PC2 (11.9%)
10
0
−10
−20
−30
−25
0
25
PC1 (75.5%)
d
soma+neurites (filter top)
neurites only (filter bottom)
proteins from somata+neurites
proteins from neurites only
Western Blot
e
somata+neurites
neurites only
bio.repl.: 1 2 3 1 2 3
RIOK2
Rsl24d1
Nucleolin
75kDa
20kDa
15kDa
100kDa
f
protein levels in neurites only
(relative to the soma+neurites comparment) [%]
100
75
50
25
0
**
**
Nucleolin
RIOK2
Rsl24d1

Suppl. Fig. 1

Suppl. Figure 1. Proteomic characterization of neuronal subcellular compartments. (**a**) Bar plot of the number of quantified protein groups in the total lysate across biological replicates of each subcellular compartment. The bars related to the somata+neurites compartment are colored in dark green, the ones for neurites only in light green. (**b**) Hierarchical clustering of biological replicates according to protein levels in lysates from somata+neurites or neurites compartments. Cells are color coded according to Pearson correlation coefficients. (**c**) PCA analysis showing similarities across protein levels in lysates from somata+neurites (dark green) or neurites only (light green) compartments. The variability explained by each Principal Component (PC) is shown in brackets. (**d**) Schematic of the experimental design. Proteins were purified from either subcellular compartment (somata+neurites vs neurites only) and measured by Western Blot. (**e**) Detection of protein of interest across subcellular compartments by Western Blot (three biological replicates). (**f**) Boxplot of the intensity levels of the indicated proteins in the neurite-only compartment, normalized to the soma+neurites compartment (data shown in f). Each dot represents an independent biological replicate. Anova, p = 5.7e−05; *t*-test between Nucleolin and RIOK2, p = 0.0093; *t*-test between Nucleolin and Rsl24d1, p = 0.0033.

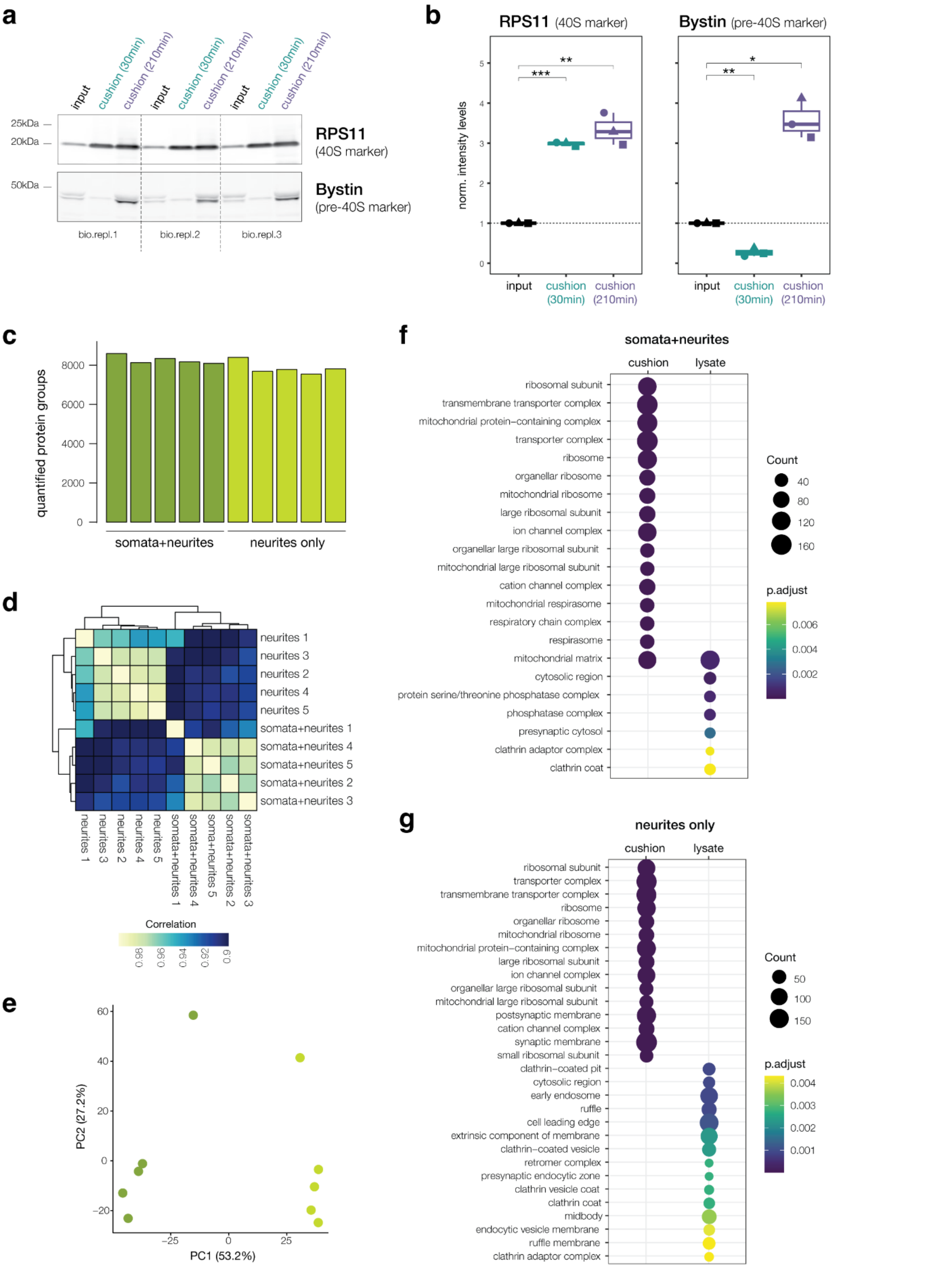
a
input
cushion (30min)
cushion (210min)
25kDa
20kDa
50kDa
RPS11
(40S marker)
Bystin
(pre-40S marker)
bio.repl.1
bio.repl.2
bio.repl.3
b
RPS11 (40S marker)
Bystin (pre-40S marker)
norm. intensity levels
input
cushion (30min)
cushion (210min)
c
quantified protein groups
somata+neurites
neurites only
d
neurites 1
neurites 3
neurites 2
neurites 4
neurites 5
somata+neurites 1
somata+neurites 4
somata+neurites 5
somata+neurites 2
somata+neurites 3
Correlation
e
PC2 (27.2%)
PC1 (53.2%)
f
somata+neurites
cushion
lysate
ribosomal subunit
transmembrane transporter complex
mitochondrial protein−containing complex
transporter complex
ribosome
organellar ribosome
mitochondrial ribosome
large ribosomal subunit
ion channel complex
organellar large ribosomal subunit
mitochondrial large ribosomal subunit
cation channel complex
mitochondrial respirasome
respiratory chain complex
respirasome
mitochondrial matrix
cytosolic region
protein serine/threonine phosphatase complex
phosphatase complex
presynaptic cytosol
clathrin adaptor complex
clathrin coat
Count
p.adjust
g
neurites only
cushion
lysate
ribosomal subunit
transporter complex
transmembrane transporter complex
ribosome
organellar ribosome
mitochondrial ribosome
mitochondrial protein−containing complex
large ribosomal subunit
ion channel complex
organellar large ribosomal subunit
mitochondrial large ribosomal subunit
postsynaptic membrane
cation channel complex
synaptic membrane
small ribosomal subunit
clathrin−coated pit
cytosolic region
early endosome
ruffle
cell leading edge
extrinsic component of membrane
clathrin−coated vesicle
retromer complex
presynaptic endocytic zone
clathrin vesicle coat
clathrin coat
midbody
endocytic vesicle membrane
ruffle membrane
clathrin adaptor complex
Count
p.adjust

Suppl. Fig. 2

Suppl. Figure 2. Proteomic characterization of sucrose cushioning from different neuronal subcellular compartments. (**a**) Western blot of the cleared lysates (input, 3% of the total volume) or cushion samples (50% of the total volume). (**b**) Quantification of the Western blot analysis. While the core Ribosomal Protein RPS11 was significantly enriched through sucrose cushioning both after 30 min and 210 min long centrifugations, the Ribosome Biogenesis Factor Bystin was significantly enriched only after 210 min long centrifugation. Each dot represents a biological replicate. For RPS11, Anova, $p = 3.7e{-}05$; *t*-test between input and 30min cushion, $p = 0.00019$; *t*-test between input and 210min cushion, $p = 0.0097$. For Bystin, Anova, $p = 2.1e{-}05$; *t*-test between input and 30min cushion, $p = 0.0061$; *t*-test between input and 210min cushion, $p = 0.012$. Both the global p-value (Anova test, on the top) and the p-value for each comparison (t-test) between the input and the cushion samples are shown. Abbreviations: small ribosomal subunit (40S), biological replicate (bio.repl.). (**c**) Bar plot of the number of quantified protein groups in the cushion samples across biological replicates of each subcellular compartment. The bars related to the somata+neurites compartment are colored in dark green, the ones for neurites only in light green. (**d**) Hierarchical clustering of biological replicates according to protein levels in cushion samples from somata+neurites or neurites compartments. Cells are color coded according to Pearson correlation coefficients. (**e**) PCA analysis showing similarities across protein levels in cushion samples from somata+neurites (dark green) or neurites only (light green) compartments. The variability explained by each Principal Component (PC) is shown in brackets. (**f**-**g**) Gene Ontology (GO) analysis of Cellular Components terms overrepresented ($FDR<0.01$) among the differentially regulated proteins between total lysates and cushion samples from either somata+neurites (**f**) or neurites-only (**g**).

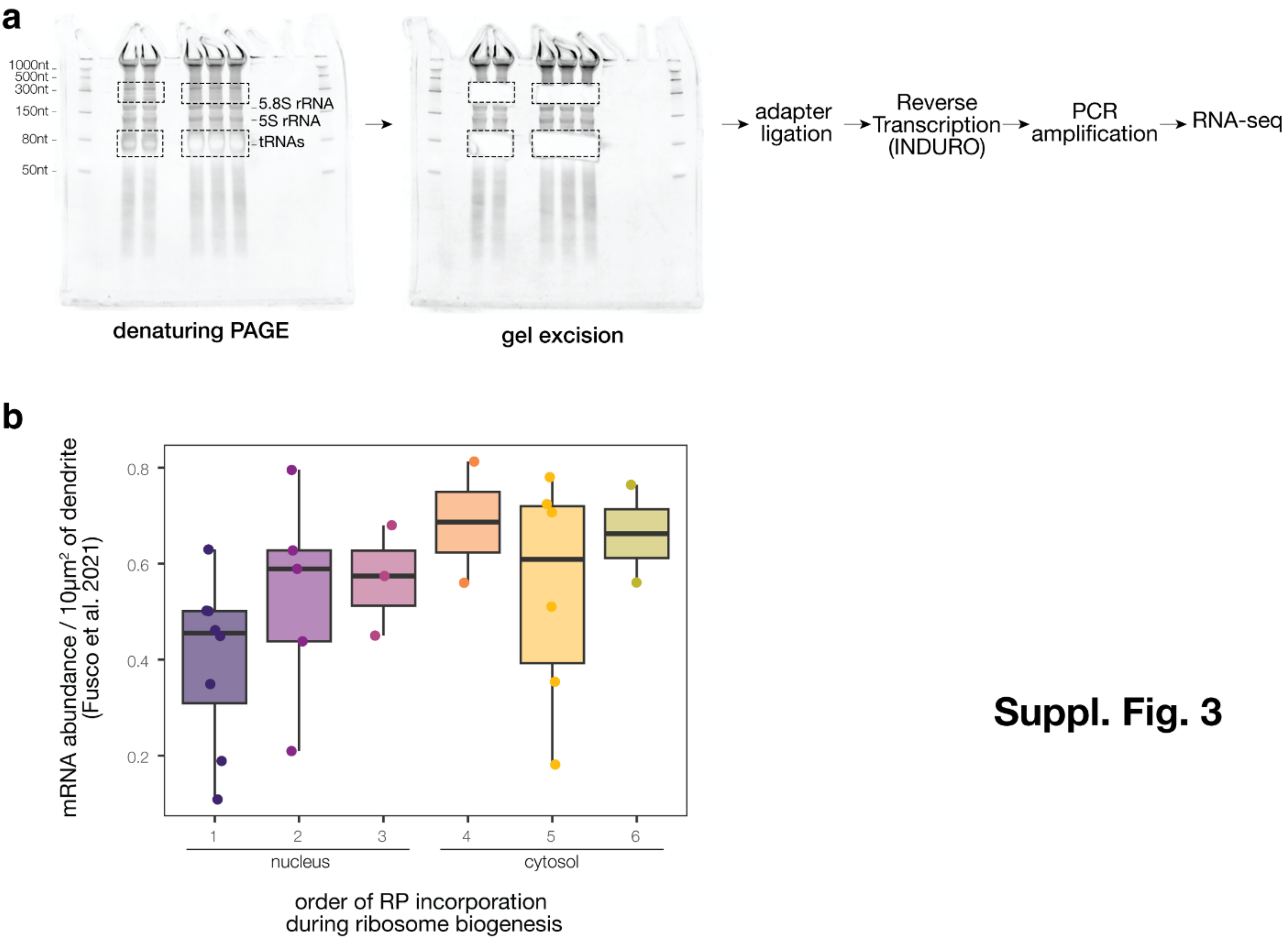
a
1000nt
500nt
300nt
150nt
80nt
50nt
5.8S rRNA
5S rRNA
tRNAs
denaturing PAGE
gel excision
adapter ligation
Reverse Transcription (INDURO)
PCR amplification
RNA-seq
b
mRNA abundance / 10µm² of dendrite (Fusco et al. 2021)
0.8
0.6
0.4
0.2
1
2
3
4
5
6
nucleus
cytosol
order of RP incorporation during ribosome biogenesis

Suppl. Fig. 3

Suppl. Figure 3. Customized pre-rRNA-seq pipeline and RP mRNAs localization in dendrites. (**a**) Schematic of the pipeline for the pre-rRNA-seq experiments. After running the purified RNA samples in a denaturing gel, RNA species of interest are enriched by gel excision. For the pre-rRNA samples, gels are cut between 160 and 400nt. Samples are then processed for adapter ligation and the Reverse Transcription, both of which introduce a 3'end bias. In particular, the Reverse Transcriptase INDURO is used because it is better able to by-pass secondary structures. After PCR amplification, samples are prepared for RNA sequencing. (**b**) Box plot of the mean RP mRNA abundance per 10μm^2 of dendrite (as measured in Fusco et al. 2021) according to the order and site of RP incorporation during ribosome biogenesis. Each dot represents a Ribosomal Protein.

Supplementary Table 1. Manually curated list of Ribosome Biogenesis Factors. List of rat ribosome biogenesis factors, compiled from the literature. The following publications were particularly instrumental for the curation of this list: Bohnsack and Bohnsack, 2019 (1); Dörner et al., 2023 (2); Nerurkar et al., 2020 (3); Prattes et al., 2019 (4). We indicate here the number of biological replicates of total lysates from either compartment (total replicates = 5) where the corresponding gene was identified. Protein intensity values of each replicate are shown in Suppl. Table 2. Additionally, we note that some of these RBFs were previously identified in axonal preparations from mice (Poulopoulos et al., 2019 (5)), rats (Chuang et al., 2018 (6)) and frogs (Cagnetta et al., 2018 (7), Shigeoka et al., 2019 (8)). Abbreviations: small ribosomal subunit (40S), large ribosomal subunit (60S), Nucleus (N), Nucleolus (Nu), Nucleoplasm (NP), Cytoplasm (C).

Gene name	Ribosomal subunit involved in	Location of first interaction	Location of last interaction	Strength of interaction	Known extra-ribosome biogenesis function?	Number of Soma replicates with gene detected	Number of Neurites replicates with gene detected	Identified in other publications
Abce1	40S	C	C	transient	yes	5	5	Poulopoulos et al., 2019; Cagnetta et al., 2018; Shigeoka et al., 2019
Bop1	60S	Nu	NP	stable	no	5	3	
Bysl	40S	N	C	stable	no	5	5	
Csnk1d	40S	N	C	stable	yes	5	5	
Csnk1e	40S	N	C	stable	yes	5	5	Chuang et al., 2018
Ddx18	60S	Nu	Nu	stable	yes	5	5	
Dimt1	40S	N	C	stable	no	5	0	
Dusp12	60S	N	C	stable	yes	5	5	
Eif6	60S	N	C	stable	yes	5	5	Chuang et al., 2018; Cagnetta et al., 2018
Fbl	40S	Nu	Nu	stable	yes	5	5	Poulopoulos et al., 2019; Chuang et al., 2018
Ftsj3	40S	Nu	Nu	stable	yes	5	0	Chuang et al., 2018
Gtpbp4	60S	NP	C	stable	no	5	0	Shigeoka et al., 2019
Gtpbp4	60S	NP	C	stable	no	5	5	Shigeoka et al., 2019
Llph	60S	Nu	C	stable	yes	5	0	
Lsg1	60S	C	C	transient	no	5	5	
Ltv1	40S	NP	C	stable	no	5	4	
Mak16	60S	Nu	NP	transient	no	5	1	
Mdn1	60S	Nu	NP	stable	no	5	0	
Mdn1	60S	Nu	NP	stable	no	5	1	
Mdn1	60S	Nu	NP	stable	no	5	3	
Mphosph10	40S	Nu	N	stable	no	5	2	
Mrto4	60S	N	C	stable	no	5	5	
Nifk	60S	Nu	N	stable	no	5	1	Chuang et al., 2018
Nle1	60S	NP	C	stable	yes	5	5	
Nmd3	60S	N	C	stable	no	5	5	
Nob1	40S	N	C	stable	no	5	5	
Nop53	60S	Nu	Nu	stable	yes	5	0	
Nsa2	60S	Nu	C	stable	no	5	5	
Nvl	60S	Nu	NP	transient	yes	5	0	
Pdcd11	both	Nu	N	stable	no	5	5	
Pelo	40S	C	C	transient	yes	5	5	Chuang et al., 2018
Pes1	60S	Nu	NP	stable	no	5	5	Chuang et al., 2018
Pno1	40S	N	C	stable	no	5	5	
Pno1	40S	N	C	stable	no	1	0	
Rae1	60S	N	C	stable	yes	5	5	
Rbm28	60S	Nu	Nu	stable	yes	5	1	
Riok1	40S	C	C	stable	yes	5	5	
Riok2	40S	N	C	stable	no	5	5	
Riok3	40S	C	C	NA	yes	5	5	
Rpf1	60S	Nu	NP	transient	no	4	0	
Rpf1	60S	Nu	NP	transient	no	5	0	
Rrp1	60S	Nu	NP	transient	no	5	0	
Rrp12	40S	Nu	C	stable	no	5	5	
Rsl24d1	60S	Nu	C	stable	no	5	0	
Sbds	60S	C	C	transient	no	5	5	
Spata5	60S	C	C	transient	yes	5	5	
Spata5l1	60S	C	C	transient	yes	4	4	
Tsr1	40S	NP	C	stable	no	5	5	
Wdr12	60S	Nu	NP	stable	no	5	5	
Wdr37	60S	Nu	NP	stable	yes	5	5	
Wdr74	60S	Nu	Nu	stable	yes	5	1	
Xrn1	40S	C	C	transient	yes	5	5	
Zfp593	60S	Nu	C	stable	yes	5	1	
Zfp622	60S	C	C	stable	yes	5	5	

Supplementary Table 2. List of antibodies.

Target	Provider	Identifier	Dilution
Bystin	Kutay lab		1:2500 (WB)
Eri1	abcam	ab236945	1:100 (IF)
GFAP	abcam	ab7260	1:2000 (IF)
MAP2	SYSY	188004	1:2000 (IF)
Nob1	ThermoFisher	A304-680A-T	1:100 (IF)
Nucleolin	abcam	ab22758	1:2000 (WB)
PSD95	ThermoFisher		1:1000 (WB)
RIOK2	ThermoFisher	H00055781-B01P	1:200 (WB)
RPS11	Bethyl	A303-936A	1:2500 (WB)
Rsl24d1	Santa Cruz Biotechnology	sc-100840	1:200 (WB)
Goat anti-guinea pig Dylight405	Jackson ImmunoResearch	106-475-003	1:1000 (IF)
Goat anti-guinea pig-Alexa488	ThermoFisher	A11073	1:1000 (IF)
Goat anti-mouse-Alexa594	ThermoFisher	A11005	1:1000 (IF)
Goat anti-mouse-Alexa488	ThermoFisher	A11001	1:1000 (IF)
Goat anti-rabbit-Alexa594	ThermoFisher	A11037	1:1000 (IF)
Goat anti- rabbit-Alexa488	ThermoFisher	A11008	1:1000 (IF)
Goat anti-rabbit IRDye 680	LICOR	926-68071	1:5000 (WB)
Goat anti-mouse IgG IRDye 680	LICOR	926-68020	1:5000 (WB)
Goat anti-mouse IRDye 800	LICOR	926-32210	1:5000 (WB)
Goat anti-rabbit IRDye 800	LICOR	926-32211	1:5000 (WB)

Supplementary Table 3. Sequence of Primers and gBlocks used for molecular cloning.

Primer or gBlocks name	Sequence (5’ → 3’)
DIMT1_Forward	CATTTTGGCAAAGAATTGCTCGAGGCCACCATGCCGAAGGTCAAGTCG
DIMT1_Reverse	AATTCCCGGGGTGTTACCCGCACTACCCGCACTGGAAAAATGAATACCTTCTGCGTTG
RIOK2_Forward	CATTTTGGCAAAGAATTGCTCGAGGCCACCATGGGGAAAGTGAATGTGGC
RIOK2 _Reverse	AATTCCCGGGGTGTTACCAGCACTACCCGCACTTTCTCCCCAAAAGCTGGCT
RPS14_Forward	TTTGGCAAAGAATTGCTCGAGGCCACCATGGCACCTCGCAAGGGG
RPS14_Reverse	AATTCCCGGGGTGTTACCCGCACTACCCGCACTCAGACGGCGACCCCGA
RPS14 insert	GCCACCATGGCACCTCGCAAGGGGAAGGAAAAGAAGGAAGAACAGGTCATCAGCCTTG<br>GACCTCAGGTGGCTGAAGGAGAGAATGTATTTGGTGTCTGCCACATCTTTGCATCCTTCA<br>ATGATACCTTTGTCCATGTTACTGATCTTTCTGGCAAGGAAACCATCTGCCGAGTAACTG<br>GTGGAATGAAGGTGAAGGCTGACCGAGATGAGTCCTCTCCGTATGCAGCCATGTTGGCT<br>GCCCAGGATGTGGCCCAGAGGTGCAAGGAACTGGGCATCACTGCCCTGCATATCAAAC<br>TCCGGGCCACAGGAGGAAACAGGACCAAGACCCCTGGACCTGGAGCCCAGTCAGCCCT<br>CAGAGCTCTTGCTCGCTCTGGGATGAAGATTGGGCGGATTGAGGATGTCACCCCCATCC<br>CCTCTGACAGCACTCGAAGGAAGGGTGGTCGTCGGGGTCGCCGTCTG
Pescadillo (gBlocks Gene Fragment)	TGCCCAGCCAGGTGTGGAGTGCGGGTAGTGCGGGTGGAGGCCTTGAGAAGAAGAAGT<br>ATGAACGAGGCTCGGCCACCAACTACATCACCCGGAACAAAGCCCGGAAGAAGCTCCA<br>GCTGAGCTTGGCTGACTTTAGGCGGCTGTGCATTCTGAAGGGCATTTATCCCCATGAAC<br>CCAAACACAAGAAGAAGGTTAACAAGGGTTCTACAGCAGCCCGAACGTTTTACCTTATCA<br>AAGACATCAGGTTTCTCCTCCACGAACCCATTGTCAACAAGTTCCGTGAATACAAGGTGT<br>TCGTCCGGAAGCTCCGGAAGGCTTATGGGAAGAGCGAGTGGAACACTGTAGAGCGTTT<br>AAAGGACAATAAGCCCAACTACAAACTCGACCACATCATCAAGGAACGGTATCCCACGT<br>TCATCGATGCCCTGCGGGACCTGGACGATGCCCTCTCCATGTGCTTCCTGTTTTCCACC<br>TTCCCGCGGACTGGCAAGTGCCACGTGCAGACCATTCAGCTGTGCCGCCGGCTCACTG<br>TGGAGTTCATGCACTACATTATCGCTGCCCGTGCCCTGCGCAAGGTCTTCCTGTCCATC<br>AAAGGCATTTACTACCAGGCCGAGGTACTGGGGCAGCCCATCGTGTGGATCACTCCCTA<br>TGCCTTCTCCCATGACCACCCGACAGACGTGGACTACAGGGTCATGGCCACCTTCACCG<br>AGTTCTACACCACGCTGCTGGGCTTTGTCAACTTCCGCCTTTACCAGTTGCTCAACCTCC<br>ACTATCCCCCGAAGCTCGAGGGTCAGGCCCAAGCAGAGGCAAAGGCCGGTGAGGGCA<br>CCTACGCGTTGGACTCCGAGAGTTGTATGGAGAAACTGGCAGCCCTCAGTGCCAGCCT<br>GGCCCGCGTGGTGGTGCCTGCCACAGAGGAGGAGGCCGAGGTGGATGAGTTTCCCAC<br>CGATGGGGAGATGTCAGCGCAGGAGGAAGACCGCAGGAAGGAGCTGGAGGCGCAGGA<br>GAAGCACAAGAAGCTTTTTGAGGGCCTGAAGTTCTTCCTGAACCGAGAGGTGCCCCGTG<br>AGGCCCTGGCCTTCATCATCAGGAGTTTTGGTGGGGAAGTGTCCTGGGACAAATCTTTG<br>TGCATTGGGGCCACCTATGACGTCACAGACTCCCGCATCACCCATCAGATTGTCGACCG<br>GCCTGGGCAGCAGACCTCAGTCATTGGCAGGTGCTACGTGCAGCCCCAGTGGGTGTTT<br>GACTCAGTGAACGCCAGGCTCCTTCTCCCCGTGGCAGAGTACTTCTCTGGGGTGCAGCT<br>GCCCCCACACCTTTCACCCTTTGTGACCGAGAAGGAAGGAGATTACGTTCCACCTGAGA<br>AGCTGAAGCTGCTGGCTCTGCAGCGGGGAGAGGACCCAGGAAACCTGAATGAGTCAGA<br>AGAGGAGGAGGAAGAGGACGACAACAACGAAGGTGATGGTGATGAAGAGGGAGAAAAT<br>GAGGAGGAGGAGGAAGATGCAGAGGCTGGTTCAGAAAAGGAGGAAGAGGCCCGGCTG<br>GCAGCCCTGGAAGAGCAGAGGATGGAGGGGAAGAAGCCCAGGGTGATGGCAGGCACC<br>TTGAAGCTGGAGGATAAGCAGCGGCTGGCCCAGGAGGAGGAGAGTGAGGCCAAGCGC<br>CTGGCCATTATGATGATGAAGAAGCGGGAGAAGTACCTGTACCAGAAGATCATGTTTGG<br>CAAGAGGCGAAAAATCCGAGAGGCCAACAAGCTGGCGGAGAAGCGGAAAGCCCACGAT<br>GAGGCGGTGAGGTCTGAGAAGAAGGCCAAGAAGGCAAGGCCGGAGTAAGATATCCATC<br>ACACTGGCG

Dendra2 (gBlocks Gene Fragment)	ATGAACACCCCGGGAATTAACCTGATCAAGGAGGACATGCGCGTGAAGGTGCACATGG<br>AGGGCAACGTGAACGGCCACGCCTTCGTGATCGAGGGCGAGGGCAAGGGCAAGCCCT<br>ACGAGGGCACCCAGACCGCCAACCTGACCGTGAAGGAGGGCGCCCCCCTGCCCTTCA<br>GCTACGACATCCTGACCACCGCCGTGCACTACGGCAACCGGGTGTTCACCAAGTACCC<br>CGAGGACATCCCCGACTACTTCAAGCAGAGCTTCCCCGAGGGCTACAGCTGGGAGCGC<br>ACCATGACCTTCGAGGACAAGGGCATCTGCACCATCCGCAGCGACATCAGCCTGGAGG<br>GCGACTGCTTCTTCCAGAACGTGCGCTTCAAGGGCACCAACTTCCCCCCCAACGGCCC<br>CGTGATGCAGAAGAAGACCCTGAAGTGGGAGCCCAGCACCGAGAAGCTGCACGTGCGC<br>GACGGCCTGCTGGTGGGCAACATCAACATGGCCCTGCTGCTGGAGGGCGGCGGCCAC<br>TACCTGTGCGACTTCAAGACCACCTACAAGGCCAAGAAGGTGGTGCAGCTGCCCGACG<br>CCCACTTCGTGGACCACCGCATCGAGATCCTGGGCAACGACAGCGACTACAACAAGGT<br>GAAGCTGTACGAGCACGCCGTGGCCCGCTACAGCCCCCTGCCCAGCCAGGTGTGGTAA

Supplementary Methods:

Sample preparation for MS analysis. Proteins were digested using S-Traps according to an adapted version of the suspension trapping protocol described by the manufacturer (ProtiFi, Huntington, NY). Peptides were desalted using C18 StageTips (Rappsilber, Juri, 2007), dried by vacuum centrifugation and stored at -20°C until LC-MS analysis.

LC-MS/MS Analysis. Dried peptides were reconstituted in 20 µl of 95% H_2O, 5% acetonitrile (ACN) with 0.1% FA. Peptides were loaded onto a C18-PepMap 100 trapping column (particle size 3 µm, L = 20 mm, ThermoFisher Scientific) and separated on a C18 analytical column with an integrated emitter (particle size = 1.7 µm, ID = 75 µm, L = 50 cm, CoAnn Technologies) using a nano-HPLC (Dionex U3000 RSLCnano). Temperature of the column oven was maintained at 55 °C. Trapping was carried out for 6 min with a flow rate of 6 µL/min using a loading buffer (100% H_2O, 2% ACN with 0.05% TFA). Peptides were separated by a gradient of water (buffer A: 100% H_2O and 0.1% FA) and acetonitrile (buffer B: 80% ACN, 20% H_2O and 0.1% FA) with a constant flow rate of 250nL/min. Peptides were eluted by a non-linear gradient with 120 min active gradient time, as selected for the respective MS method by (9); total run including loading, washing and equilibration was 155 min. Analysis was carried out on a Fusion Lumos mass spectrometer (ThermoFisher Scientific) operated in positive polarity and data independent acquisition (DIA) mode. The DIA method defined MS1 scans followed by 40 DIA scans with optimized segment widths, as published by (9). In brief, the DIA-method had the following settings. Full scan: orbitrap resolution = 120k, AGC target = 125%, mass range = 350-1650 m/z and maximum injection time = 100 ms. DIA scan: activation type: HCD, HCD collision energy = 27%, orbitrap resolution = 30k, AGC target = 2000%, maximum injection time = dynamic. The mass spectrometry proteomics data have been deposited to the ProteomeXchange Consortium via the PRIDE (10) partner repository with the dataset identifier PXD058169.

MS-data processing. DIA raw files were processed with the open-source software DIA-NN (version 1.8.2 beta 27) using a library-free approach. The predicted library was generated using the in silico FASTA digest (Trypsin/P) option with the UniProtKB database (Proteome_ID: UP000002494) for *Rattus norvegicus*. Deep learning-based spectra- and RT-prediction was enabled. The covered peptide length range was set to 7-30 amino acids, missed cleavages to 2 and precursor charge range to 1-5. Methionine oxidation and protein N-terminal acetylation were set as variable modifications. Cysteine carbamidomethylation was selected as a fixed modification. The maximum number of variable modifications per peptide was limited to 1. According to most of DIA-NN's default settings, MS1 and MS2 accuracies as well as scan-windows were set to 0, isotopologues were enabled, while match-between-runs and shared spectra were disabled. Protein inference was performed using genes with the heuristic protein inference option enabled. The neural network classifier was set to single-pass mode and the quantification strategy was selected as "QuantUMS (high precision)". The cross-run normalization was set to "RT-dependent", the library generation to "smart profiling", the speed and Ram usage to "optimal results".

MS Post-processing and statistical analysis. The DIA-NN report table was imported in the statistical computing software R. The entries were filtered for 1% FDR at protein (Global.PG.Q.Value and PG.Q.Value) and precursor level (Q.Value) as well as for unique peptides (Proteotypic) before performing the protein roll-up of the precursor intensities using the diann_maxlfq function provided by the DIA-NN R package (https://github.com/vdemichev/DiaNN). For further analysis, protein intensities were log2-scaled and normalized using median-centering. For the differential expression analysis, only genes with at least 7 observations across replicates were considered.
GO overrepresentation analysis was performed using the ClusterProfiler R package (https://github.com/YuLab-SMU/clusterProfiler). In brief, gene lists of regulated proteins were compared to a custom background dataset containing all genes identified in the experiment. The analysis was then carried out using gene symbols, the ontology setting "cellular compartment", Benjamini-Hochberg

FDR correction and an FDR cut-off of 1%. Redundant GO terms were simplified according to the adjusted p-value with a redundancy cut-off of 0.7 before merging the enrichment results for visualization of the top-25 terms per condition.

Gel electrophoresis for Northern Blot. Samples (1µg of RNA) were added to a volume of the same size of 2X RNA Loading Dye (NEB, B0363A). RNAs were denatured at 70 °C for 10 min and returned to ice before being loaded on a denaturing Novex 10% TBE-Urea Gels (ThermoFisher, EC6875BOX) in 1X TBE running buffer at 200V for 90 minutes. Total RNA was visualized with SYBR Gold Nucleic Acid Gel Stain (ThermoFisher, S11494).

Northern Blot transfer and crosslink. Northern Blot procedure was performed as previously described (11). Briefly, RNA was transferred to an Ambion BrightStar-Plus membrane (ThermoFisher, AM10100) at 30V for 2 hours at 4 °C, using a semi-dry transfer cell (BioRad, 170-3957). Membrane was washed in 2x SSC and then placed on a Whatmann paper soaked in 0.753 grams of 1-ethyl-3-(3-dimethylaminopropyl) carbodiimide (EDC; Sigma, 7750) dissolved in 1.25 M 1-Methylimidazole (Sigma, M50834) pH 8.0. To facilitate RNA–membrane cross-linking, the membrane was incubated at 60 °C for 2 hours. Residual cross-linking reagents were removed by thoroughly rinsing the membrane with distilled water.

Probes generation for Northern Blot. Probes with the following sequence were purchased as DNA oligos from IDT:

- ITS2-site4 (GGG+CGGCG+ATTGA+TCGTC+AAGCGA, where + indicates LNA base)
- SNORD104 (GATTCGCATCACCCGGATCAGCAGTCTAACGC)
- mature 5.8S (GGCCGCAAGTGCGTTCGAAGTGTCGATGAT).

To generate DIG containing probes, the DIG Oligo tailing kit (Roche, 3353583910) was used. Briefly, 2µL containing 50pmol of probes were mixed with RNase free water (7µL), reaction buffer (4µL), CoCl2-solution (4µL), DIG-dUTP solution (1µL), dATP solution (1µL) 400 U Terminal transferase (1µL), and incubate for 15 min at 37 °C. The reaction was stopped by adding 2µL 0.2M EDTA (pH 8.0).

Northern Blot hybridization and detection. Membranes were pre-hybridized in 10 mL of ULTRAhyb Ultrasensitive Hybridization Buffer (ThermoFisher, AM8670) for at least 30 min at 42 °C. Probes were heated at 95 °C for 1 min and then added to the prehybridized blot at a final concentration of 0.5nM overnight (for ITS2-site4 and SNORD104) or 0.025nM for 30 min (for mature 5.8S). Blots were washed twice for 5 min at 42 °C in 2X SSC, 0.1% SDS, twice for 15 min at 42 °C in 0.1X SSC , 0.1% SDS, and once for 5 min at room temperature in 1x DIG Washing Buffer from the DIG Wash and Block Buffer Set (Roche, 11585762001). Membranes were incubated for 3 hours at room temperature in 50 mL of 1x DIG Blocking buffer in 1x Maleic Acid Buffer. Anti-Digoxigenin-AP, Fab fragments (Roche, 11 093 274 910) were then added (1:10,000) and the membranes were incubated at room temperature for 30 min. Membranes were then washed twice for 15 min in 1x DIG Washing Buffer, and once for 5 min in 1x DIG detection buffer. CSPD (Roche, 11 655 884 001) was then diluted 1:100 in 1x DIG detection buffer and applied to the membrane. Blots were inserted in a Blot Development folder (Azure Biosystems, AC2126) and incubated protected from light at room temperature for 5 min, and 37 °C for 10 min. Membranes were kept protected from light at room temperature for at least 30 minutes prior to imaging. Stripping was performed following the protocol available online from the Drummond lab. Briefly, membranes were incubated twice for 15 min in 50 mL of boiling 0.1X SSC, 0.5% SDS, before starting with the hybridization and detection steps for the new probe.

Northern Blot analysis. Image acquisition was performed at the Azure using the chemiluminescence program. Band intensity was quantified with AzureSpot Pro (v1.0 – 366, Azure Biosystems). Image analysis was then performed in R.

References

1. K. E. Bohnsack, M. T. Bohnsack, Uncovering the assembly pathway of human ribosomes and its emerging links to disease. *Embo J* **38**, e100278 (2019).

2. K. Dörner, C. Ruggeri, I. Zemp, U. Kutay, Ribosome biogenesis factors—from names to functions. *EMBO J.* **42**, e112699 (2023).

3. P. Nerurkar, *et al.*, Eukaryotic ribosome assembly and nuclear export*. International Review of Cell and Molecular Biology* **319**, 107-140 (2015).

4. M. Prattes, Y.-H. Lo, H. Bergler, R. E. Stanley, Shaping the Nascent Ribosome: AAA-ATPases in Eukaryotic Ribosome Biogenesis. *Biomol* **9**, 715 (2019).

5. A. Poulopoulos, *et al.*, Subcellular transcriptomes and proteomes of developing axon projections in the cerebral cortex. *Nature* **565**, 356–360 (2019).

6. C.-F. Chuang, C.-E. King, B.-W. Ho, K.-Y. Chien, Y.-C. Chang, Unbiased Proteomic Study of the Axons of Cultured Rat Cortical Neurons. *J. Proteome Res.* **17**, 1953–1966 (2018).

7. R. Cagnetta, C. K. Frese, T. Shigeoka, J. Krijgsveld, C. E. Holt, Rapid Cue-Specific Remodeling of the Nascent Axonal Proteome. *Neuron* **99**, 29-46.e4 (2018).

8. T. Shigeoka, *et al.*, On-Site Ribosome Remodeling by Locally Synthesized Ribosomal Proteins in Axons. *CellReports* **29**, 3605-3619.e10 (2019).

9. J. Muntel, *et al.*, Comparison of Protein Quantification in a Complex Background by DIA and TMT Workflows with Fixed Instrument Time. *J. Proteome Res.* **18**, 1340–1351 (2019).

10. Y. Pérez-Riverol, *et al.*, The PRIDE database and related tools and resources in 2019: Improving support for quantification data. *Nucleic acids research* **47**, D442–D450 (2019).

11. S. W. Kim, *et al.*, A sensitive non-radioactive northern blot method to detect small RNAs. *Nucleic acids research* **38**, e98–e98 (2010).
